# Flavones and Related Compounds: Synthesis and Biological Activity

**DOI:** 10.3390/molecules28186528

**Published:** 2023-09-08

**Authors:** Denisa Leonte, Daniel Ungureanu, Valentin Zaharia

**Affiliations:** Department of Organic Chemistry, Iuliu Hațieganu University of Medicine and Pharmacy, Victor Babeş 41, RO-400012 Cluj-Napoca, Romania; leonte.denisa@umfcluj.ro (D.L.); daniel.ungureanu@elearn.umfcluj.ro (D.U.)

**Keywords:** chalcones, flavones, flavonols, aurones, anticancer activity, antimicrobial activity

## Abstract

This review focuses on the synthesis and biological activity of flavones and their related flavonoidic compounds, namely flavonols and aurones. Among the biological activities of natural and synthetic flavones and aurones, their anticancer, antioxidant, and antimicrobial properties are highlighted and detailed in this review. Starting from the structures of natural flavones acting on multiple anticancer targets (myricetin, genkwanin, and other structurally related compounds), new flavone analogs were recently designed and evaluated for their anticancer activity. The most representative compounds and their anticancer activity are summarized in this review. Natural flavones recognized for their antimicrobial properties (baicalein, luteolin, quercetol, apigenin, kaempferol, tricin) have been recently derivatized or structurally modulated by chemical synthetic methods in order to obtain new effective antimicrobial flavonoidic derivatives with improved biological properties. The most promising antimicrobial agents are systematically highlighted in this review. The most applied method for the synthesis of flavones and aurones is based on the oxidative cyclization of *o*-hydroxychalcones. Depending on the reaction conditions and the structure of the precursor, in some cases, several cyclization products result simultaneously: flavones, flavanones, flavonols, and aurones. Based on the literature data and the results obtained by our research group, our aim is to highlight the most promising methods for the synthesis of flavones, as well as the synthetic routes for the other structurally related cyclization products, such as hydroxyflavones and aurones, while considering that, in practice, it is difficult to predict which is the main or exclusive cyclization product of *o*-hydroxychalcones under certain reaction conditions.

## 1. Introduction

Flavonoids are a widely distributed group of natural polyphenolic compounds that are found in plants usually in glycosylated form and have been shown to possess a wide range of biological activities, including antioxidant, anti-inflammatory, antibacterial, antiviral, and anticancer properties, making them an attractive target for synthesis and further study.

Structurally, flavonoids are functional aromatic compounds constituted by a C6-C3-C6 structure. The bioprecursor of flavonoids is the amino acid L-phenylalanine, which is transformed into phenyl-propenoyl-*S*-CoA with the involvement of the phenylalanine ammonia-lyase enzyme. Enzymatic condensation of phenyl-propenoyl-*S*-CoA with three malonyl-*S*-CoA units, followed by cyclization, yields *o*-hydroxychalcones that are structurally 1,3-diarylpropen-1-ones [1]. 

The reactive α,β-unsaturated ketone structure and the presence of hydroxy groups in *o*-hydroxychalcones make their cyclization possible, resulting in different flavonoidic compounds. Similar to biochemical cyclization pathways, in organic synthesis, the cyclization of *o*-hydroxychalcones represents the most useful way to obtain compounds from aurones, flavanones, flavones, flavonols, and flavylium salts, as will be detailed in Section 3.

The outstanding biological potential of natural flavonoids has attracted interest in the medical field, meaning that many of their synthetic analogs are currently known as promising candidates in treatments for cancer; microbial, fungal, and viral infections; inflammatory diseases; and diabetes.

Among the flavonoidic compounds, flavones and flavonols are related by the fact that they possess the same basic skeleton, the 2-phenyl-chromen-4-one system [1]. Flavones represent one of the most studied sub-class of flavonoids due to their wide distribution in plants and their wide structural diversity. 

**Figure 1:**
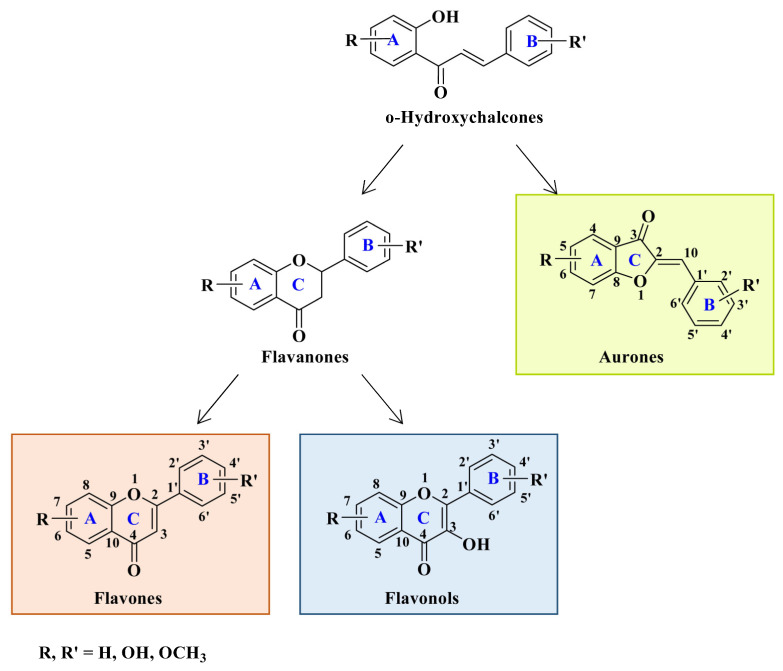
General structures of flavones, flavonols, aurones, and their common precursors, the *o*-hydroxychalcones.

Flavonols, also called hydroxyflavones, differ from flavones by the presence of a hydroxy group at position 3 in the chromen-4-one ring (C ring, Figure 1) [1]. Although they have very similar structures, natural flavonols are not formed from chalcones via flavones as intermediates but through another biochemical pathway, with the involvement of other enzymes, via flavanones. Flavanones are common bioprecursors for flavones and flavonols [2].

Aurones, 2-benzylidenebenzofuran-3(2*H*)-ones [2], also belong to the flavonoid class, being structural isomers of flavones (Figure 1). Even if aurones are less known compared to flavones, research on them has experienced significant development in recent years due to their promising therapeutic potential. 

Because of their related structure, flavones, flavonols, and aurones have common properties, such as the interesting way they exert their antioxidant, anticancer, antimicrobial, and other pharmacological activities [2].

In recent years, various methods have been developed for synthesizing flavones and related compounds, mainly hydroxyflavones and aurones, including chemical, biochemical, enzymatic, and total synthesis. Chemical synthesis is based on different approaches involving the use of different precursors or reagents, with the earliest developed synthesis methodologies for flavones emanating from the late 1890s–1900s (the von Kostanecki methodology and von Auwers synthesis [3]). 

This review aims to present the most relevant methods for flavone and aurone synthesis, starting with the earliest and concluding with the recent methods. We consider it important to specify some examples from the literature and from our own research in which aurones and hydroxyflavones were obtained via the cyclization of *o*-hydroxychalcones, considering that they are structurally related compounds and that sometimes it is difficult to predict which would be the main or exclusive reaction product under certain reaction conditions.

Although the synthesis methodologies were first applied for obtaining the basic skeleton of natural flavones or aurones (in which rings A and B are benzene rings), some of these methods were successfully extended to obtain new bioactive flavonoidic analogs of natural compounds or flavone/aurone hybrids through structural modulations at the level of the A, B, or C rings with the aim of obtaining new bioisosters with improved biological functions. All structural modulations retain the aromatic character of the A and B rings, which is essential for their biological activity.

The structural modulations made on the A ring of flavones and aurones involve grafting different electron-withdrawing (halogen atoms, cian, or nitro) or electron-donating substituents such as hydroxy, methoxy, or acyloxy groups. Bulkier substituents such as pyperidine, directly connected to the A ring or through a linker such as benzyloxy, benzylamino, isopentyloxy, or benzylaminomethylene, are also introduced at different positions of the A ring of some flavonoidic analogs with reported anticancer/antimicrobial activity, as exemplified below.

The structural modulations at the aromatic B ring include the introduction of various electron-withdrawing (halogen atoms, carbamoyl, or trifluoromethyl groups) or electron-donating substituents (hydroxy, methoxy, benzyloxy, alkyl, acylamino, alkylamino, dialkylamino, or other multifunctionalized residues such as amino acid residues). Other structural modifications are based on replacing the B ring with other pentaatomic or hexaatomic aromatic heterocycles such as thiazole, pyrazole, thiophene, and pyridine, alone or linked/condensed with other (hetero)aromatic rings in order to obtain extended π-conjugated aromatic systems such as 2-phenylthiazole, thiazolo [3,2-*b*][1,2,4]triazole, 1,3-diphenylpyrazole, 3-naphtyl-1-phenylpyrazole, and quinoline.

The structural changes at the level of the C ring of flavones and aurones have been less frequently investigated. The most frequently reported changes include the derivatization of the hydroxy group of hydroxyflavones via alkylation or acylation. Recent attempts to obtain azaaurones, compounds which contain nitrogen as a heteroatom in the C ring instead of oxygen, have been made. It was found that replacing the intracyclic oxygen of aurones with nitrogen is beneficial for selective cytotoxicity against some multidrug resistant cancer cells, such as the resistant cancer cell line P-glycoprotein-overexpressing human doxorubicin resistant uterine sarcoma cells (MES-SA/Dx5) [4].

## 2. Biological Activity of Flavones, Flavonols, and Aurones

### 2.1. Anticancer Activity

The antitumor activity of flavones is most often due to their ability to target certain key structures that lead to cell cycle arrest and the apoptosis of tumor cells. Thus, flavones can inhibit the specific enzymes responsible for tumorigenesis, which are normally involved in the regulation of the cell cycle but whose function is deregulated under pathological conditions, for example, protein kinase C (PKC) [5], cyclin-dependent kinases (CDK) [6], casein kinases (CK) [7], PIM-1 kinases [8], death-associated protein kinase 1 (DAPK-1), and tyrosine kinases [9]. Some flavones can inhibit the polymerization of tubulin, thus preventing the formation of microtubules [10]. All this leads to cell cycle arrest, most often in the G2/M phase. Flavones can also activate certain enzymes that cause tumor cell apoptosis, such as caspases [11,12].

Natural flavones such as apigenin and nobiletin can regulate the expression of important inflammatory signaling pathways, including nuclear factor erythroid 2-related factor 2 (Nrf2) and the nuclear factor kappa-light-chain-enhancer of activated B cells (NF-κB). The antioxidant properties of several natural flavones are attributable to their ability to regulate the expression of Nrf2/heme oxygenase-1 (HO-1), which decreases free radical levels and oxidative stress [13]. Nuclear factor erythroid 2-related factor (Nrf2) can interact with the NF-κB signaling pathway to maintain cellular redox homeostasis during inflammatory states. As the NF-κB pathway activates the expression of genes implicated in inflammation that can lead to chronic inflammation, tumor development, or proliferation, the Nrf2 pathway displays important antioxidant roles, such as mediating the release of ROS (reactive oxygen species) induced by NF-κB or suppressing the transcription of NF-κB-dependent pro-inflammatory genes [14]. Thus, the activation of Nrf2 pathway will suppress the NF-κB pathway and reduce TNFα, IL-6, and IL-1β proinflammatory cytokine levels [14].

The potential of flavones to act on multiple anticancer targets or by synergic mechanisms of action allows them to be considered as key structures for the development of new multitarget-acting therapeutic agents.

**Figure 2:**
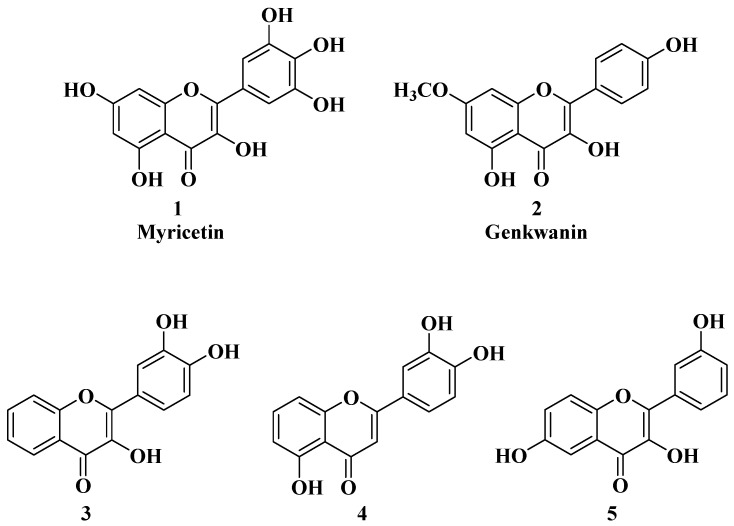
Polyphenolic flavones with anticancer and antioxidant activity [24,27].

In several cases, the anticancer activity of natural flavones and aurones is closely related to their antioxidant activity. Myricetin (Figure 2), a natural flavone with polyphenol structure, presents good antioxidant properties by acting as a scavenger for reactive oxygen species and by enhancing the activity of glutathione-*S*-transferase [15]. Myricetin also presents great antitumor properties by targeting key structures, leading to cell cycle arrest and apoptosis. Myricetin has been shown to inhibit several enzymes involved in cell cycle regulation whose functions were deregulated under pathological conditions, namely, PKC, CK2, PIM-1, and DAPK1 [16]. Myricetin promotes tumor cell apoptosis by modulating certain signaling pathways, including Bcl2 (B-cell lymphoma 2), NF-κB, MAPKs (mitogen-activated protein kinases), and the Wnt/β-catenin signaling pathway [16,17,18]. Recently, it was reported that myricetin inhibits interferon-γ-induced programmed death ligand-1 (PD-L1) and indoleamine 2,3-dioxygenase 1 (IDO1) expression in lung cancer cells via the regulation of the Janus kinase/signal transducer and activator of the JAK/STAT-IRF1 transcription pathway [19]. According to the authors of [19], in their study, Myricetin recovered the function of T cells in the lung cancer cells and Jurkat-PD-1 T cells. Myricetin restored the survival, proliferation, CD69 expression, and interleukin-2 (IL-2) secretion of Jurkat-PD-1 T cells suppressed by IFN-γ-treated lung cancer cells [19]. PD-L1 and ISO1 are two immune checkpoints responsible for the immune escape of tumors. Thus, as an inhibitor of IFN-γ-induced PD-L1 and ISO1, myricetin has potential applications in tumor immunotherapy.

Recent studies have shown that myricetin induces apoptosis and autophagy in human gastric cancer cells through the inhibition of the PI3K/Akt/mTOR pathway (phosphoinositide 3-kinase, PI3K/Protein kinase B, Akt/Mechanistic target of rapamycin, mTOR) [20]. The abnormal increase in the activity of the PI3K/Akt/mTOR pathway is associated with various malignancies; therefore, the modulation of this signaling pathway represents a new strategy, in particular in gastric cancer treatment [21].

Myricetin also proved to be effective in preventing mutagenesis induced by different carcinogenic compounds such as formaldehyde [22]. Myricetin alleviates the formaldehyde-enhanced Warburg effect in tumor cells through the inhibition of human hypoxia-inducible factor 1 subunit alpha (HIF-1α), an important target in lung and ovarian tumors [22].

Gu Ling et al. recently revealed that myricetin regulates the p38 MAPK pathway by targeting MAP Kinase Kinase 3 (MKK3) in non-small cell lung cancer cells (NSCLC) [23]. These results encourage future research on the development of new anticancer agents, MKK3 inhibitors, through the structural modulation of myricetin.

Genkwanin (Figure 2), another natural flavone with antioxidant properties, has demonstrated promising anticancer activity against a series of cancer cell lines, including human MCF-7 breast cancer (IC_50_ = 13.6 ± 0.3 µg/mL), HepG-2 human hepatocellular carcinoma (IC_50_ = 22.5 ± 0.3 µg/mL), and HCT-116 colon cancer (IC_50_ = 15.4 ± 0.5 µg/mL). Genkwanin is also able to reduce the migration, invasion, and proliferation of lung cancer cells by targeting the phosphoinositide 3-kinase (PI3K) and phospho-protein kinase B (AKT) signaling pathways [24]. Due to this mechanism, genkwanin represents an effective option for the treatment of cancer proliferation and metastasis. Because genkwanin presents low oral bioavailability, genkwanin nanosuspensions were prepared in order to improve its solubility and pharmacokinetic profile. Li Y et al. reported the therapeutic potential of genkwanin nanosuspensions as novel antitumor agents in breast carcinoma therapy [25].

Spiegel M. et al. established that, through the bond dissociation enthalpy (BDE) of the hydrogen atom transfer (HAT) mechanism, the antioxidant activity of flavones could be related to the presence of a hydroxy group located on the B ring, especially in position C4′, more than the A-ring substitution. Regarding flavonols, the presence of a hydroxy group in C3 is beneficial for their antioxidant activity. These positions present the lowest values of bond dissociation entalphy (BDE = 84.4 kcal/mol for C4′, in the case of luteolin, and BDE = 84.6 kcal/mol for C3, in the case of morin) [26].

The anticancer activity of flavones could be correlated with their antioxidant activity, but it is not a mandatory rule in all cases. Grigalius I. and Petrikaite V. studied the relationship between the anticancer and antioxidant activities of trihydroxyflavones. The antioxidant activity was evaluated by using the DPPH (2,2-diphenyl-1-picrylhydrazyl) method, and the anticancer activity was evaluated by using the MTT (3-[4,5-dimethylthiazol-2-yl]-2,5-diphenyl tetrazolium bromide) method, both of which were performed on three different types of human cancer cell lines: lung (A549), breast (MCF-7), and brain epithelium (U87). Based on the calculation of the Pearson coefficient (r), a moderate correlation was revealed between the two biological properties [27]. It was found that the substituents on the phenyl ring (B ring) are the most important for the antioxidant activity of trihydroxyflavones. Thus, the most potent antioxidants have the *o*-dihydroxy group (catechol) on the B ring and are involved in binding hydroxy, peroxyl, and peroxynitrile radicals [27] (Figure 2, compounds **3** and **4**). However, hydroxyflavone **5** does not possess this structural feature, but it does present the best anticancer activity, thus, in this case, alluding to the existence of other mechanisms of action for anticancer activity besides the neutralizing effect of free radicals.

Zhao L. et al studied the structural elements of flavones capable of blocking different serine-threonine kinases involved in the cell cycle. Structure–activity relationship studies were conducted for PKC, CK-2, PIM-1 kinase, DAPK-1, and CDK. It was found that the hydroxy groups grafted on rings A, B, and C act as H-bond donors/acceptors in the interaction with PKC, PIM-1, DAPK-1, and CDK [28]. For the inhibition of CK-2, it was found that the presence of the halogen atoms Br and Cl at positions 6 and 8 of the A ring, respectively, and the hydroxy group only in position 4 of the B ring are beneficial. The carbonyl group located in position 4 of the chromen-4-one ring acts as a H-bond acceptor in the interaction with various amino acid residues from CK-2, CDK, and PIM-1. The benzene ring (B ring) interacts by π–π stacking with the phenylalanine residue Fen113 of CK-2, and through this mechanism, it also blocks the ATP binding site of these enzymes. The benzene ring (B ring) can also make van der Waals interactions with certain hydrophobic residues from CDK-9 and PIM-1, thus making additional contact with these enzymes without blocking the binding of ATP. It has also been observed that changing the position of the phenyl ring from 2 to 3, specific to isoflavones, leads to the loss of activity [28].

Flavopiridol (Table 1, line 1) is a semisynthetic flavone that is currently being used in clinical trials as an anticancer agent for the treatment of acute myeloid leukemia. This compound acts by inhibiting kinases CDK-1, -2, -4, -6, and -7, all of which are competitive with ATP. At the same time, flavopiridol significantly inhibits kinases CDK-9 (non-competitive with ATP) [9,28]. Flavopiridol also inhibits the activity of positive transcription elongation factor (P-TEFb), a cyclin-dependent kinase controlling elongation via RNA polymerase II [29].

The anticancer activity of flavopiridol is due to the presence of a chromone moiety that is bioisosteric with the purine ring of ATP and binds competitively to the ATP binding pocket of CDK. The benzene ring (ring B) provides additional contact with the enzyme, as it interacts with different regions than those occupied by ATP, participating in van der Waals-type interactions with other amino acid units [28]. Other important elements for the inhibition of kinase activity by flavopiridol are the hydroxy groups at C-7 and C-5, the carbonyl group at C-4, the nitrogen atom, and the hydroxy group from the piperidine, and all of these functional groups are involved in the formation of hydrogen bonds with CDK [28].

Starting from the structures of two natural products with anticancer activity with different mechanisms of action, 3,5,4′-trimethoxystilbene and 5,6,7-trimethoxyflavone, Hassan A.H. et al. synthesized new antiproliferative compounds by combining two pharmacophore moieties in the same molecule by replacing the vinylene residue in stilbene with the amide group [30]. The cytotoxic activities of the synthesized compounds against several cancer cell lines were determined at 10 μM doses in all cases. The structures of the most active compounds are presented in Table 1, lines 2–4.

Flavone–stylbene hybrids in which the nitrogen atom of the amide linker is attached to the flavone moiety proved to be more citotoxic than the corresponding compounds with the opposite amide linker configuration. Trimethoxylated flavone–stylbene hybrids showed superior activity compared to dimethoxylated flavone–stylbene hybrids on hematologic, colorectal, central nervous system, ovarian, renal, and breast cancer cell lines [30]. On lung cancer cell lines, the dimethoxylated derivatives were generally more active than the trimethoxylated ones. Most of the tested hybrid compounds showed selective activity, showing no cytotoxicity on normal cells. Their anticancer mechanism of action consists of inducing apoptosis and inhibiting cell proliferation [30].

Continuing their research, Hassan A.H. et al. synthesized a series of trimethoxyflavone-based aryl-amides, starting from the structures of already approved arylamide-type medicinal compounds (imatinib, masitinib) and replacing the bulky aromatic entity in their structure with 5,6,7-trimethoxyflavone and 5-hydroxy-6,7-dimethoxyflavone. The formation of the amide bond was carried out in the 3′ and 4′ positions on the B ring of the flavone using 3′-amino and 4′-amino precursors coupled with various acyl chlorides and 3′-carboxyl precursors condensed with aryl amines, respectively [31].

Two flavones presented good broad-spectrum anticancer activity by triggering cell cycle arrest in the G1 phase (Table 1, line 5). These compounds could represent hit compounds for the design of new, more potent inhibitors of STE20/GCK-IV kinase family members, including HGK, TNIK, and MINK1 kinases. It was found that the presence of the carbonyl of the amide linker attached to the flavone moiety is beneficial for the anticancer activity of the tested flavone-based aryl-amides. Reversing the attachment mode of the amide linker led to a significant decrease in anticancer activity [31].

**Figure 3:**
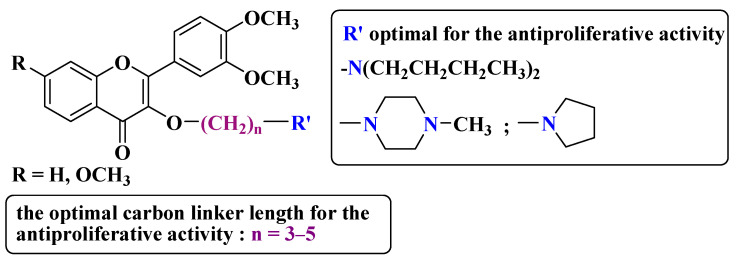
3-*O*-substituted flavonols reported as anti-prostate cancer agents. Structure–activity relationship study [32].

A series of dimethoxyflavonols and trimethoxyflavonols derivatives were obtained via the alkylation of the hydroxy group at position 3 of the chromen-4-one ring (C ring) (Figure 3). The compounds were investigated for their anticancer activity on both androgen-sensitive (LNCaP) and androgen-insensitive (PC-3 and DU145) prostate cancer cell lines [32].

It was found that the alkylation of the hydroxy group in position 3 generally increased the antiproliferative activity of the compounds. The presence of an amino group linked to the hydroxy group at position 3 of the flavonols through a three- to five-carbon linker is beneficial for antiproliferative activity against the three human prostate cancer cell lines with tumor selectivity. *N*-methylpiperazin-1-yl, pyrrolidin-1-yl, and dibutyl amino groups proved to be beneficial, improving the anticancer activity of the tested compounds. The most promising derivative in terms of selectivity, anticancer activity, and bioavailability contains a dibutyl amino group linked to the oxygen at position 3 via a three-carbon linker (Table 1, line 6). The bioavailability of the tested compounds was superior to that of fisetin [32].

Starting from a series of differently substituted chalcones, Pontes et al. synthesized a series of chromene–chalcone hybrid compounds in order to test their anticancer activity on breast cancer cell lines. The most active compound is depicted in Table 1, line 7. The mechanism of action involves the inhibition of cell migration and induction of apoptosis, by determining cell cycle arrest in the G2/M phase. Moreover, this compound has been proved to alter tubulin polymerization, representing a promising new microtubule-destabilizing agent. It was found that the presence of the halogen atoms grafted on the basic skeleton of chromene–chalcone hybrids is beneficial to antitumor activity. Brominated compounds presented superior activity to chlorinated and fluorinated compounds. The evaluated compounds presented selective cytotoxicity on cancer cell lines compared to non-cancerous cell lines [10].

New hybrid compounds of flavones (chrysin and kaempferol) and substituted 1,2,3-triazoles were recently synthesized via the chemical derivatization of the hydroxyl groups of chrysin and kaempferol with functionalized 1,2,3-triazole compounds [33]. The antitumor activity of the obtained mono- and bis-coupled hybrids was evaluated in vitro on 60 cell lines of 9 common cancer types (NCI60) [33]. The hybrid compounds presenting the most significant antiproliferative effect are mentioned in Table 1, lines 8, 9.

A series of new heterocyclic derivatives were recently synthesized via the functionalization of a flavone ring with an aminophenoxy moiety in different positions of the A ring and a phenoxy moiety in different positions of the B ring [34]. Their cytotoxicity was investigated in vitro against two human non-small cell lung cancer (NSCLC) cell lines (A549 and NCI-H1975). It was found that the presence of a 4-aminophenoxy group at the sixth position of the A ring and a terminal phenoxy group on the B ring is beneficial for cancer-selective cytotoxicity. A flavone derivative containing a phenoxy moiety at the C’3 position of the B ring and a *p*-aminophenoxy group at the sixth position of the A ring was the most effective, presenting micromolar IC_50_ values (for A549 and H1975) and a high selectivity index (SI > 10, Table 1, line 10). Further flow cytometric analyses showed that this compound induces apoptosis and cell cycle arrest in the G2/M phase through the up-regulation of p21 expression [34]. The absence of the phenoxy moiety on the B ring and the different position of the *p*-aminophenoxy moieties on the A ring decreased the efficacy and selectivity of aminophenoxy derivatives [34].

New *C*-dimethylated flavones were recently synthesized and evaluated for their anti-tubercular and anticancer activity [35]. In this study, four flavones presented anticancer activity against a human adenocarcinoma A549 cell line, with IC_50_ values between 39 and 48 μM (Table 1, lines 11–14). This study’s in silico docking simulations revealed that these four compounds present improved binding and interaction profiles against the epidermal growth factor receptor (EGFR) [35].

Other recently reported examples of synthetic flavones with antitumor activity are illustrated in Table 1, lines 15–20.

Natural and synthetic aurones possess a broad variety of biological activities, including antiproliferative activity against different cancer cell lines. The anticancer activity of aurones is due to their ability to interact with different key antitumor molecular targets, and examples of such interactions include the following: the inhibition of serine/threonine cyclin-dependent kinases (CDK 1 and 2) [36], the inhibition of topoisomerase IIα [37], the inhibition of sphingosine kinase (SphK) [38], and interfering with microtubule assembly [39]. In some cases, it was found that the anticancer activity of aurones is strongly related to their antioxidant activity [40]. 

Several aurones have been shown to modulate the activity of ATP-dependent efflux pumps such as P-glycoprotein [41] and breast cancer resistance protein (BCRP/ABCG2) [42]. Through this mechanism, aurones can potentiate the effect of simultaneously administered anticancer chemotherapeutics by blocking the multidrug resistance mechanisms of tumor cells.

Our research group synthesized a series of aurone analogs by replacing the B ring (phenyl) with the 2-arylthiazole system in order to obtain compounds with superior anticancer activity, considering the anticancer potential of thiazole derivatives. Two aurone analogs were active against cancer cell lines resistant to currently used chemotherapeutics, such as multidrug-resistant leukemia cell lines and breast cancer cell lines, and both showed cytotoxic activities that were superior to doxorubicin (Table 1, lines 21, 22) [43]. Other recently reported examples of synthetic aurones with antitumor activity are illustrated in Table 1, lines 23–31.

**Table 1:** Synthetic analogs of flavones and aurones with antitumor properties.

Entry	Chemical Structure	Cancer Cell Lines against the Tested Compounds Present Cytotoxic Activity	Ref.
1	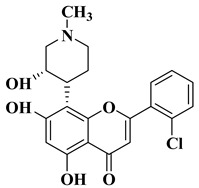 Flavopiridol	- Acute myeloid leukemia cells U266 (69% growth inhibition in G0/G1 cell cycle, dosage: 100 nM).	[44]
2	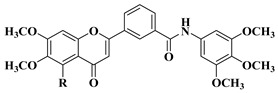	R = OCH_3_ - Cell lines of hematologic cancers RPMI8226 (99.70%), CCRFCEM (68.74%), HL60(TB) (64.01%), K562 (72.38%), MOLT4 (89.58%), SR (70.19%), growth inhibition determined at 10 µM dosage. - Non-small-cell lung cancer (NSCLC) A549 (56.48%), HOP62 (62.50%), HOP92 (75.26%), H226 (41.45%), H23 (51.21%), H460 (69.96%), H522 (65.28%), growth inhibition determined at 10 µM dosage. - Breast cancer cells MCF7 (65.73%), HS578T (90.80%), BT549 (73.02%), MDAMB468 (52.58%), growth inhibition determined at 10 µM dosage.	[30]
		R = OH - Ovarian cancer cell lines OVCAR3 (76.24%), OVCAR8 (76.91%), ADRRES (50.62%) at 10 µM dosage. - Breast cancer cells MCF7 (50.65%), HS578T (103.91%), BT549 (93.00%), MDAMB468 (40.13%), growth inhibition determined at 10 µM dosage.	
3	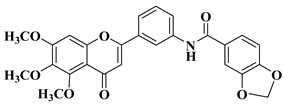	- Cell lines of hematologic cancers RPMI8226 (153.74%), CCRFCEM (111.94%), HL60(TB) (65.43%), K562 (82.60%), MOLT4 (97.10%), SR (88.49%), growth inhibition determined at 10 µM dosage. - Non-small-cell lung cancer cell lines (NSCLC) HOP92 (106.11%), H322M (56.46%), H460 (72.81%), H522 (63.27%), growth inhibition determined at 10 µM dosage.	[30]
4	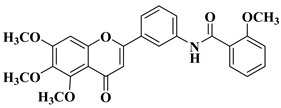	- Cell lines of hematologic cancers RPMI8226 (93.25%), CCRFCEM (83.98%), HL60(TB) (42.34%), K562 (63.61%), MOLT4 (71.17%), growth inhibition determined at 10 µM dosage. - Non-small-cell lung cancer cell lines (NSCLC) A549 (51.79%), HOP92 (100.39%), H322M (56.40%), H522 (55.74%), growth inhibition determined at 10 µM dosage.	[30]
5	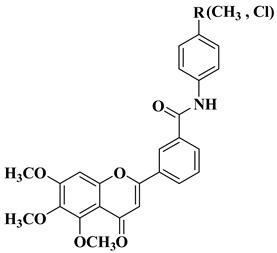	- Large spectra antitumor activity: melanoma (160.26–107.81% SKMEL5), hematologic (111.12–92.74% leukemia HL60), renal (129.05% RXF393), colon (98.27–82.03% COLO205), lung (93.28% H522), brain (147.04–141.63% SF295 glioma), ovarian (76.54–51.79% IGROV1, OVCAR3, OVCAR8, ADREES, SKOV3), growth inhibition determined at 10 µM dosage.	[31]
6	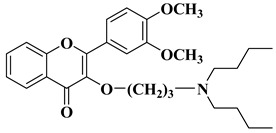	- Androgen-sensitive prostate cancer cell line LNCaP (IC_50_ = 2.4 ± 1.5 µM). - Androgen-insensitive prostate cancer cell lines PC-3 (IC_50_ = 1.4 ± 0.2 µM) and DU145 (IC_50_ = 7.6 ± 2.4 µM).	[32]
7	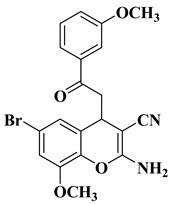	- Breast cancer cell lines MCF-7 (IC_50_ = 3.65 ± 0.021 µM), Hs578T (IC_50_ = 4.52 ± 0.019 µM), with tumor selectivity compared to non-cancer cell lines MCF-10A (4.17 and 3.08).	[10]
8	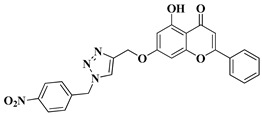	- Non-small cell lung cancer cell lines: HOP-62 (GI_50_ = 3.76 µM), HOP-92 (GI_50_ = 4.43 µM), NCI-H226 (GI_50_ = 3.51 µM), NCI-H23 (GI_50_ = 7.70 µM), NCI-H522 (GI_50_ = 6.60 µM). - Colon cancer cell line: HCT-116 (GI_50_ = 5.91 µM). - Central nervous system cancer cell lines: SF-268 (GI_50_ = 4.32 µM), SF-539 (GI_50_ = 5.17 µM), SNB-19 (GI_50_ = 4.51 µM), SNB-75 (GI_50_ = 3.74 µM). - Melanoma: MALME-3M (GI_50_ = 5.06 µM), SK-MEL-2 (GI_50_ = 6.80 µM). - Ovarian cancer cell lines: OVCAR-8 (GI_50_ = 3.76 µM), NCI/ADR-RES (GI_50_ = 5.57 µM), SK-OV-3 (GI_50_ = 6.57 µM). - Renal cancer cell lines: 786-0 (GI_50_ = 9.26 µM), ACHN (GI_50_ = 6.23 µM), CAKI-1 (GI_50_ = 5.76 µM), RXF 393 (GI_50_ = 3.58 µM). - Breast cancer cell lines: HS 578T (GI_50_ = 6.26 µM), BT-549 (GI_50_ = 7.98 µM).	[33]
9	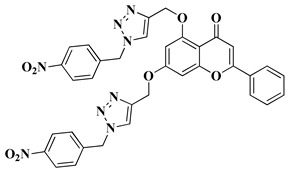	- Non-small cell lung cancer cell lines: HOP-62 (GI_50_ = 2.33 µM), HOP-92 (GI_50_ = 1.89 µM), NCI-H226 (GI_50_ = 2.07 µM), NCI-H23 (GI_50_ = 3.70 µM), NCI-H522 (GI_50_ = 3.66 µM). - Colon cancer cell line: HCT-116 (GI_50_ = 3.52 µM). CNS cancer: SF-268 (GI_50_ = 3.52 µM), SF-295 (GI_50_ = 2.32 µM), SF-539 (GI_50_ = 2.21 µM), SNB-19 (GI_50_ = 4.55 µM), SNB-75 (GI_50_ = 1.69 µM), U251 (GI_50_ = 2.80 µM). - Melanoma: MALME-3M (GI_50_ = 2.03 µM), SK-MEL-2 (GI_50_ = 4.49 µM). - Ovarian cancer cell line: IGROV1 (GI_50_ = 4.45 µM). - Renal cancer cell lines: 786-0 (GI_50_ = 1.96 µM), RXF 393 (GI_50_ = 1.78 µM), TK-10 (GI_50_ = 3.01 µM). - Breast cancer cell lines: MDA-MB-231/ATCC (GI_50_ = 2.34 µM), HS 578T (GI_50_ = 3.28 µM), MDA-MB-468 (GI_50_ = 1.97 µM).	[33]
10	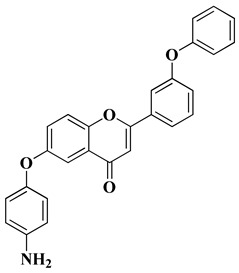	- Non-small cell lung cancer cell lines: A549 (IC_50_ = 4.2 ± 0.4 µM), NCI-H1975 (IC_50_ = 2.3 ± 0.2 µM).	[34]
11	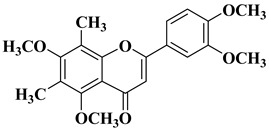	- Human adenocarcinoma cell line A549 (IC_50_ = 39.17 µM).	[35]
12	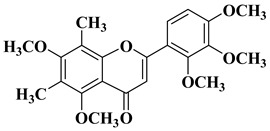	- Human adenocarcinoma cell line A549 (IC_50_ = 39.21 µM).	[35]
13	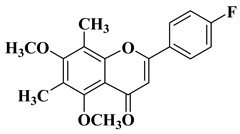	- Human adenocarcinoma cell line A549 (IC_50_ = 48.43 µM).	[35]
14	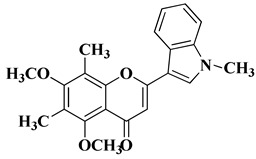	- Human adenocarcinoma cell line A549 (IC_50_ = 43.48 µM).	[35]
15	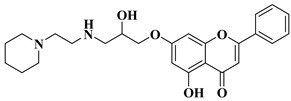	- AR-negative castration-resistant prostate cancer cell line (CRPC) as topoisomerase II catalytic inhibitor (88.9% growth inhibition at 20 µM) and by intercalating and binding to the DNA minor groove (IC_50_ = 0.13 ± 0.007 µM). - Sensitizes AR-positive CRPC cells to enzalutamide and taxanes.	[45]
16	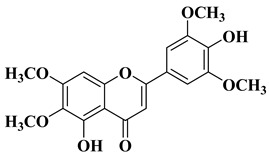	- Human pancreas adenocarcinoma ascites metastasis Aspc-1 cancer cell lines (IC_50_ = 5.30 µM).	[46]
17	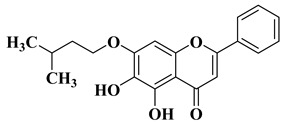	- MCF-7 breast cancer cells (IC_50_ = 5.6 ± 1.94 µM) and yeasts expressing human caspase-7.	[11]
18	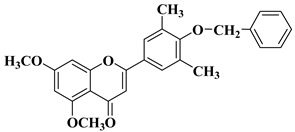	- Human erythroleukemia cell line HEL (IC_50_ = 9.945 ± 0.930 µM). - Prostate cancer cell line PC3 (IC_50_ = 6.473 ± 0.811 µM).	[47]
19	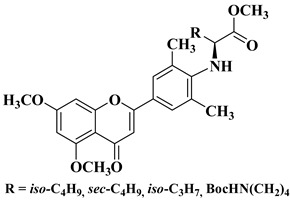	- Human erythroleukemia cell line HEL (IC_50_ = 7.563–8.886 µM). - Prostate cancer cell line PC3 (IC_50_ = 9.140–10.242 µM).	[47]
20	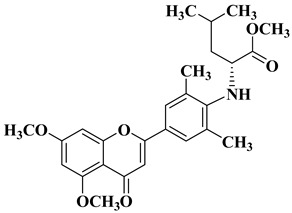	- Human erythroleukemia cell line HEL (IC_50_ = 10.526 ± 0.992 µM). - Prostate cancer cell line PC3 (IC_50_ = 11.266 ± 0.971 µM).	[47]
21	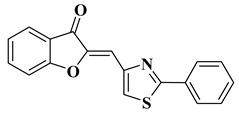	- Leukemia cell line, doxorubicin-resistant phenotype CEM/ADR5000 (IC_50_ = 5.85 ± 0.46 µM).	[43]
22	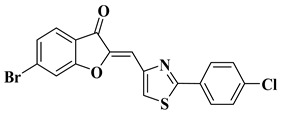	- Breast adenocarcinoma cell line, resistant phenotype MDA-MB231/BCRP (IC_50_ = 5.43 ± 3.17 µM).	[43]
23	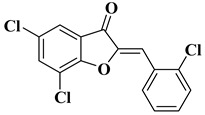	- Human colorectal cancer cell line HCT 116 (IC_50_ = 36 µM). - Human chronic myelogenous leukemia cell line K562 (IC_50_ = 23 µM). - Hormone-dependent breast cancer cell line MCF-7 (IC_50_ = 23 µM).	[48]
24	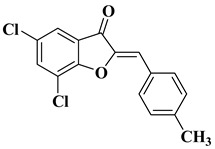	- Human chronic myelogenous leukemia cell line K562 (IC_50_ = 20 µM).	[48]
25	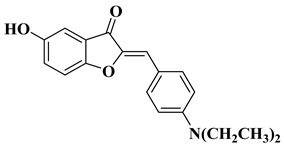	- Inhibition of in vitro angiogenesis of HUVEC (human umbilical vein endothelial cells) proliferation, motility, and tube formation (IC_50_ = 0.25 µM). - Anti-proliferative and anti-invasive activities against A549 (non-small cell lung cancer cell line, IC_50_ = 1.25 µM), and MCF-7 (breast cancer cell line, IC_50_ = 1.81 µM).	[49]
26	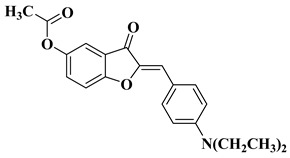	- Inhibition of in vitro angiogenesis of HUVEC (human umbilical vein endothelial cells) proliferation, motility, and tube formation (IC_50_ = 0.23 µM). - Anti-proliferative and anti-invasive activities against A549 (non-small cell lung cancer cell line, IC_50_ = 1.29 µM), and MCF-7 (breast cancer cell line, IC_50_ = 2.95 µM).	[49]
27	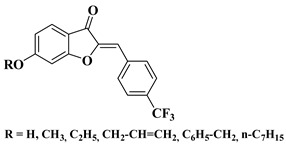	- Leucocythemia cell line HL-60 (IC_50_ = 1.54–3.53 µM). - Colorectal adenocarcinoma cell line HT-29 (IC_50_ = 4.12–8.90 µM).	[50]
28	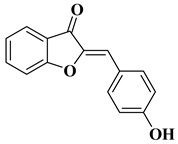	- Human oral squamous carcinoma cell lines Ca9-22 (derived from gingival tissue, CC_50_ = 37 µM), HSC-2 (CC_50_ = 57 µM), and HSC-4 (derived from tongue, CC_50_ = 31 µM), with tumor-specificity in comparison to oral normal cells.	[51]
29	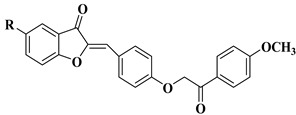	R = Cl: leukemia cell lines MOLT-4 (−17.79% mean growth percentage), and SR (−22.38% mean growth percentage). R = H: renal cancer cell line UO-31 (−44.36% mean growth percentage). The mean growth percentages were determined for five concentrations ranging from 10^−4^ to 10^−8^ M.	[52]
30	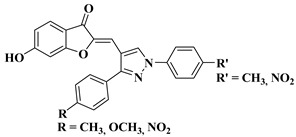	- Gastric cancer cell line CRL-1739. R′ = CH_3_ and R = CH_3_, OCH_3_, NO_2_ (IC_50_ = 25–28.3 µM). R′ = NO_2_ and R = NO_2_ (IC_50_ = 25.1 µM).	[53]
31	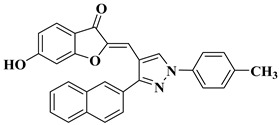	- Gastric cancer cell line CRL-1739 (IC_50_ = 27.0 µM).	[53]

### 2.2. Antibacterial and Antifungal Activity

Bacterial and fungal resistance to existing antibiotics is a worldwide health issue, particularly affecting the immunocompromised patients. Without effective antimicrobial agents, several medical procedures could endanger the lives of patients by increasing the risk of microbial infections. The basic structure of natural flavones and aurones have inspired researchers to develop new antimicrobial agents with improved bioavailability and antibacterial and antifungal properties.

Recently, it was reported that the natural flavone myricetin (Figure 2) presents anti biofilm activity against *Staphylococcus aureus* and attenuates osteomyelitis by inhibiting the Toll-like receptor-2 (TLR2)/mitogen-activated protein kinase (MAPK) pathway in experimental mice [54].

Ashok D. et al. synthesized new flavonol analogs bearing the extended heteroaromatic system 1-phenyl-3-(thiophen-2-yl)-1*H*-pyrazol-4-yl instead of a phenyl ring (B ring) and containing various substituents on the chromone system. The synthesized flavonol derivatives were screened for their antimicrobial activity against several fungal strains (*Aspergillus niger*, *Penicillium italicum*, *Fusarium oxysporum*) and bacterial strains (*Staphylococcus aureus*, *Pseudomonas aeruginosa*, *Escherichia coli*, *Bacillus subtilis*). The inhibition zones (IZ) were determined at 50 μg/mL concentration for each compound in dimethyl sulfoxide (used as a solvent). Four of the tested compounds (Table 2, lines 1–4) show good antimicrobial activity and represent hit compounds for the design of new antifungal and/or antibacterial therapeutic agents [55].

In order to obtain new flavone analogs with antibacterial activity, Lv X.H. et al. synthesized a series of flavone Mannich base derivatives by applying the Mannich reaction between primary amines and using natural flavones as components with mobile hydrogen and formaldehyde as a carbonyl component. The natural flavones used as precursors were baicalein, luteolin, quercetol, apigenin, and kaempferol. Derivatization was performed at position 8 of the chromone moiety by applying the Mannich reaction [56]. The antibacterial activity of the obtained flavone Mannich bases was evaluated for two Gram-positive bacteria (*S. aureus* and *Listeria monocytogenes*) and two Gram-negative bacteria (*E. coli* and *Salmonella gallinarium*), and novobiocin and ciprofloxacin were used as standards. The structures of the most active compounds are shown in Table 2, lines 5, 6. Through performing in vitro experiments and in silico molecular docking studies, it was found that these compounds exhibit potent inhibition against topoisomerase II and topoisomerase IV isolated from *E. coli* [56].

New hydroxyflavone derivatives containing the dimethylamino group grafted at position 4 of the benzene ring (B ring) were synthesized and evaluated for their antifungal activity against *Acremonium strictum*, *Penicillium expansum*, and *Aspergillus flavus*. Four of the tested compounds presented very good antifungal activities against some of the tested fungal strains (Table 2, lines 7–10) [57].

New quinoline-based aurone analogs were synthesized and evaluated for their antibacterial, antifungal, and anti-biofilm activity. The compounds mentioned in Table 2, lines 11–13 presented the most significant antibacterial and antifungal activities, and some of them were also shown to be good anti-biofilm agents [58].

New *C*-dimethylated flavones were recently synthesized and evaluated for their anti-tubercular and anticancer activity [35]. Two dimethylated and dimethoxylated flavones bearing the fluoro and dimethylamino substituents in position 4 of the B ring were shown to have significant antibacterial activity against the H37Rv strain of replicating *Mycobacterium tuberculosis*, with sensitivity up to 6.25 µg/mL (Table 2, line 14).

### 2.3. Antiviral Activity

Viral infections represent a global health issue and have had many implications on public health throughout history, including the appearance of new mutant viral strains and the emergence of pandemics. Specific aspects of modernization, such as rapid air transit and urbanization, have accelerated the emergence and spread of viruses. Antiviral therapy is necessary when vaccination does not bring the expected results or in the case of infections for which vaccination has not been implemented. Flavones have also been included in the research of new molecules with antiviral potential, yielding some important results and positive prospects for the future.

According to a recently reported study, the natural flavone myricetin (Figure 2) possesses potency against SARS-CoV-2 infection through blocking viral-entry facilitators and suppressing inflammation through the RIPK1/NF-κB pathway [59]. Myricetin also inhibits SASR-CoV-2 infection and replication in Vero E6 cells (EC_50_ 55.18 μM) [59]; these results suggest that this flavone represents a key structure for the design of new therapeutic agents against COVID-19.

Regarding tricin, 4′,5,7-trihydroxy-3′,5′-dimethoxyflavone, a flavone derivative with activity against cytomegalovirus (CMV), Fujimoto K.J. et al. modulated its structure by grafting a fluorine atom on the chromen-4-one ring. Thus, two compounds were obtained—6-F-tricin and 7-F-tricin—and the antiviral activity of which was measured against cytomegalovirus replicated on embryonic lung cell cultures. Compared to ganciclovir, 6-F-tricin showed much stronger activity against cytomegalovirus (Table 2, line 15). Moreover, it was observed that 6-F-tricin did not produce cytotoxicity on the used embryonic cells. Substitution with fluorine is beneficial for increasing the affinity for target proteins (in this case, for CDK9, cyclin-dependent kinase 9) [60].

The antiviral potential of flavones has also been demonstrated against tropical diseases such as Chikungunya fever. Badavath V.N. et al. synthesized nineteen flavones in order to evaluate their antiviral activity against Chikungunya virus replication. Two compounds showed activity at concentrations below 1 µg/mL (Table 2, lines 16, 17). It was observed that the more potent compounds possess heterocycles (thiophen-2-yl and pyridyn-2-yl) in position 2 of the chromen-4-one ring instead of the benzene ring (B ring). Through conducting molecular docking studies, it was deduced that these compounds act by inhibiting the Chikungunya virus protease [61].

**Table 2:** Synthetic analogs of flavones and aurones with antimicrobial (antibacterial/antifungal/antiviral) properties.

Entry	Chemical Structure	Microbial Strains against the Tested Compounds Present Antimicrobial Activity	Ref.
1	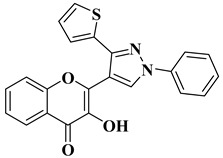	Antifungal activity (inhibition zone for 50 μg/mL solution): *Aspergillus niger* (IZ = 16 mm) *Penicillium italicum* (IZ = 20 mm) *Fusarium oxysporum* (IZ = 31 mm)	[55]
2	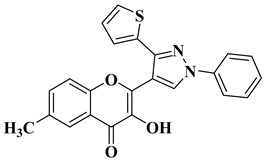	Antibacterial activity (inhibition zone for 50 μg/mL solution): *Staphylococcus aureus* (IZ = 31 mm) *Pseudomonas aeruginosa* (IZ = 11 mm) *Escherichia coli* (IZ = 30 mm)	[55]
3	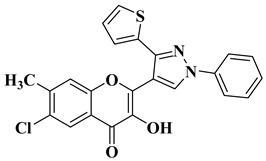	Antibacterial activity (inhibition zone for 50 μg/mL solution): *Staphylococcus aureus* (IZ = 30 mm) *Bacillus subtilis* (IZ = 11 mm) *Escherichia coli* (IZ = 31 mm) Antifungal activity (inhibition zone for 50 μg/mL solution): *Aspergillus niger* (IZ = 13 mm) *Penicillium italicum* (IZ = 24 mm) *Fusarium oxysporum* (IZ = 25 mm)	[55]
4	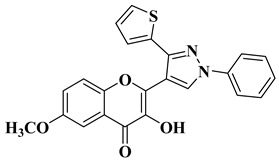	Antibacterial activity (inhibition zone for 50 μg/mL solution): *Staphylococcus aureus* (IZ = 33 mm) *Bacillus subtilis* (IZ = 17 mm) *Escherichia coli* (IZ = 33 mm) Antifungal activity (inhibition zone for 50 μg/mL solution): *Aspergillus niger* (IZ = 14 mm) *Penicillium italicum* (IZ = 26 mm) *Fusarium oxysporum* (IZ = 27 mm)	[55]
5	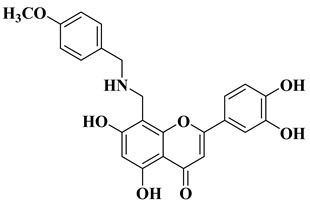	Antibacterial activity: *Staphylococcus aureus* (MIC = 2 mg/L) *Escherichia coli* (MIC = 4 mg/L) *Salmonella gallinarum* (MIC = 0.125 mg/L)	[56]
6	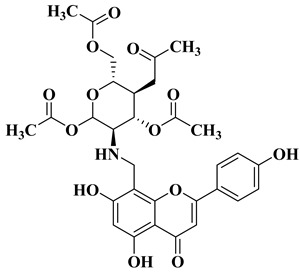	Antibacterial activity: *Staphylococcus aureus* (MIC = 1 mg/L) *Escherichia coli* (MIC = 2 mg/L) *Salmonella gallinarum* (MIC = 0.05 mg/L) *Listeria monocytogenes* (MIC = 0.5 mg/L)	[56]
7	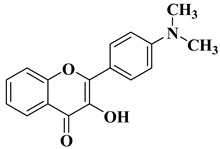	Antifungal activity (percentage inhibition at 0.25 mg/mL and, respectively 0.5 mg/mL concentration): *Acremonium strictum* (81.33%; 100%) *Penicillium expansum* (60.87%; 100%) *Aspergillus flavus* (41.02%; 65.64%)	[57]
8	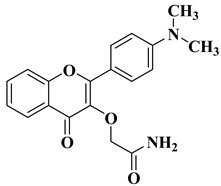	Antifungal activity (percentage inhibition at 0.25 mg/mL and, respectively 0.5 mg/mL concentration): *Acremonium strictum* (70%; 100%) *Penicillium expansum* (42.15%; 100%) *Aspergillus flavus* (6.41%; 46.15%)	[57]
9	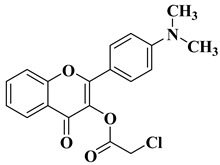	Antifungal activity (percentage inhibition at 0.25 mg/mL and, respectively 0.5 mg/mL concentration): *Acremonium strictum* (76.88%; 100%) *Aspergillus flavus* (15.38%; 60.51%)	[57]
10	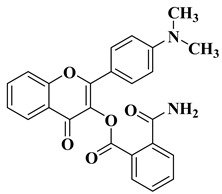	Antifungal activity (percentage inhibition at 0.25 mg/mL and, respectively 0.5 mg/mL concentration): *Acremonium strictum* (73.33%; 100%)	[57]
11	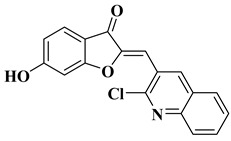	Antibacterial activity: *Staphylococcus aureus* (MIC = 1.25 mg/mL) *Bacillus subtilis* (MIC = 0.02 mg/mL) *Mycobacterium smegmatis* (MIC = 0.625 mg/mL) Antifungal activity: *Fusarium oxysporum* (MIC = 0.625 mg/mL)	[58]
12	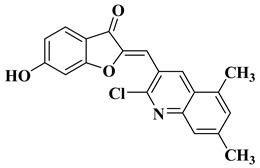	Antibacterial activity: *Staphylococcus aureus* (MIC = 2.5 mg/mL) *Bacillus subtilis* (MIC = 0.156 mg/mL) *Mycobacterium smegmatis* (MIC = 0.078 mg/mL) Anti biofilm and anti quorum sensing activity (100 μg/mL) Antifungal activity: *Fusarium oxysporum* (MIC = 0.313 mg/mL) *Candida albicans* (MIC = 0.078 mg/mL)	[58]
13	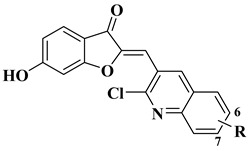	Antibacterial activity (R=6-OCH_3_, 7-Cl) *Staphylococcus aureus* (MIC = 1.25 mg/mL) *Bacillus subtilis* (MIC = 1.25 mg/mL) *Klebsiella pneumoniae* (MIC = 0.625 mg/mL) Anti biofilm activity (R=6-OCH_3_, 100 μg/mL) Antifungal activity (R=7-Cl): *Candida albicans* (MIC = 0.156 mg/mL)	[58]
14	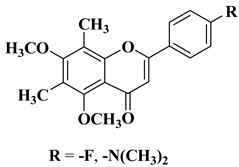	Antibacterial activity *Mycobacterium tuberculosis* H37Rv (MIC = 6.25 µg/mL)	[35]
15	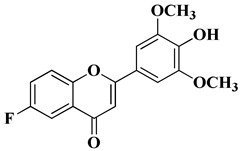	Antiviral activity *Human cytomegalovirus* (EC_50_ = 0.126 nM)	[60]
16	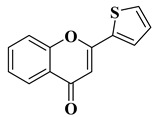	Antiviral activity *Chikungunya Virus* (IC_50_ = 0.44 µM)	[61]
17	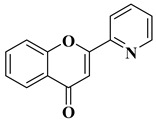	Antiviral activity *Chikungunya Virus* (IC_50_ = 0.45 µM)	[61]

## 3. Chemical Synthesis of Flavones

### 3.1. Von Kostanecki Method

**Scheme 1:**
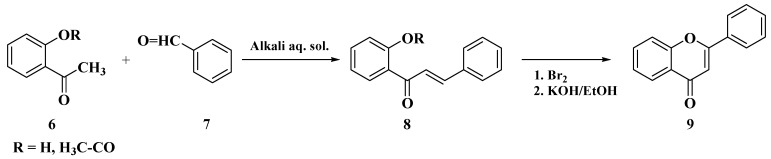
von Kostanecki method for obtaining flavones.

Stanislaus von Kostanecki’s method was established in 1898–1899 and is considered one of the earliest methods for the synthesis of flavones. It uses *o*-hydroxyacetophenone (or *o*-acetoxyacetophenone) **(6)** and benzaldehyde **(7)** as precursors to form 2′-hydroxychalcone (or 2′-acetoxychalcone) **(8)** through Claisen–Schmidt condensation. In the next step, the obtained chalcone **(8)** is converted to flavone **(9)** through bromination followed by a dehydrobromination reaction in alkali alcoholic solution (Scheme 1). 

**Scheme 2:**
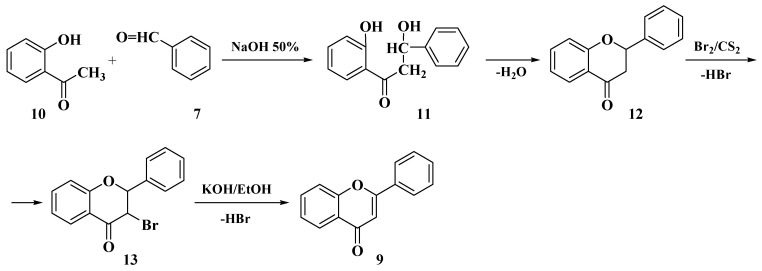
von Kostanecki method according to collaboration with Levi and Tambor.

The reaction pathway differs depending of the reaction conditions. According to von Kostanecki’s collaboration with Levi and Tambor, it is presumed that, instead of a chalcone, an aldol **(11)** between the two compounds forms; then, via cyclization, a flavanone **(12)** is formed. This flavanone is subsequently subjected to nuclear bromination with bromine in carbon disulfide, resulting in a 3-bromoflavanone **(13)** [62], and ultimately, the brominated intermediate suffers a dehydrobromination reaction, thus deriving a flavone **(9)** (Scheme 2) [63,64].

**Scheme 3:**
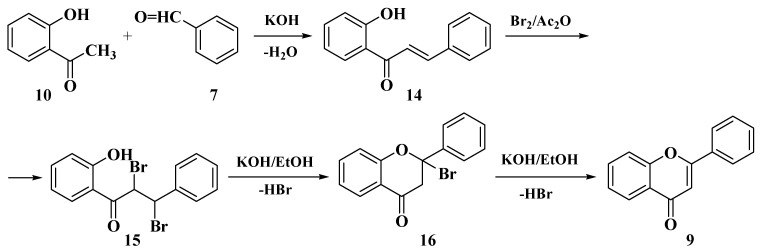
Emilewicz–von Kostanecki cyclization reaction steps.

However, according to von Kostanecki’s collaboration with Emilewicz and Tambor (also known as Emilewicz–von Kostanecki cyclization), the chalcone **(14)** is formed and then brominated, resulting in a chalcone dibromide **(15)**. This brominated compound is cyclized through the elimination of one bromine atom, resulting in a 2-bromoflavanone **(16)** and, finally, the flavone **(9)** after eliminating the second atom (Scheme 3) [3,64].

**Scheme 4:**
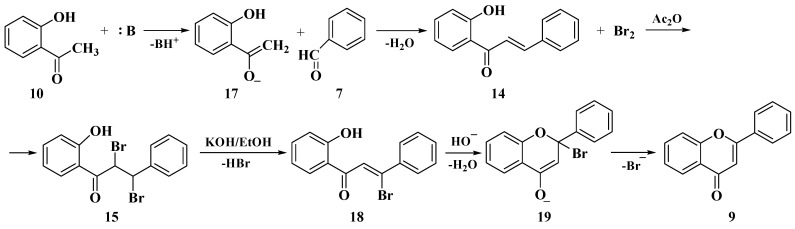
Emilewicz–von Kostanecki mechanism for the synthesis of flavones (adapted from Kshatriya et al.) [3].

The proposed mechanism starts with the Claisen–Schmidt condensation of *o*-hydroxyacetophenone **(10)** with benzaldehyde **(7)**, resulting in an *o*-hydroxychalcone **(14)**. The nucleophilic species is represented by the intermediate **(17)**, stabilized via conjugation, and formed via the deprotonation of acetophenone **(10)** in basic media. Through the bromination of the chalcone **(14)** on the C=C bond, a chalcone dibromide **(15)** is formed. Further dehydrobromination of **15** followed by cyclization of the intermediate **18** affords a 2-bromoflavanone intermediate **(19)**. The intermediate **19** leads to the flavone **9** via the expulsion of Br^−^ (Scheme 4) [3].

**Scheme 5:**
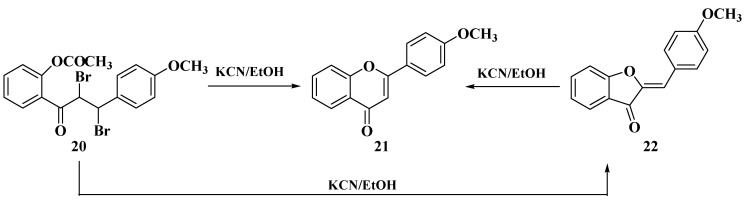
Hutchins and Wheeler method to convert chalcone dibromides and aurones to flavones using potassium cyanide in ethanol (image adapted from Fitzgerald et al.) [69].

The limitations of the von Kostanecki method are the possibility of nuclear bromination and the tendency to form 2-benzylidene-coumaran-3-ones **(22)** (benzalcoumaranones or aurones [65]) (Scheme 5) instead of flavones **(21)** when trying to synthesize natural flavones either with 5,7-disubstitution pattern, a 4′- or 5′-alkoxy substituent, or containing a phloroglucinol moiety. Better results can be obtained when methyl ether derivatives **(20)** are used as precursors [66,67,68]. Hutchins and Wheeler observed that treating the chalcone dibromides **(20)** with potassium cyanide in ethanolic solution or heating above their melting point will convert them into flavones **(21)** [68]. The same reagent converts aurones **(22)** back to flavones **(21)** (Scheme 5). The quantity of potassium cyanide influences the reaction’s outcome. In the case of 2-*p*-alkoxybenzylidenecoumaran-3-ones, refluxing with an excess of reagent will cause the ring expansion of the aurone, affording 4′-alkoxyflavones **(21)**, while treating the chalcone dibromide **(20)** with an insufficient quantity of potassium cyanide produces 2-benzylidene-coumaran-3-one (aurone **22**) instead of a flavone [69]. 

**Scheme 6:**
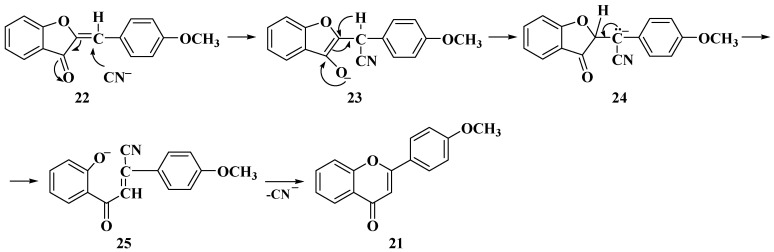
Possible mechanism for converting aurones to flavones using ethanolic potassium cyanide (image adapted from Fitzgerald et al.) [69].

The possible mechanism of obtaining flavones from aurones using ethanolic potassium cyanide starts with a nucleophilic attack by a cyanide anion on the methine carbon of the aurone **(22)**, followed by hydrogen transfer and carbanion **(24)** formation. The carbanion’s electrons migrate, recreating the double bond and preparing the ring expansion. Conjugation and intramolecular nucleophilic attack by the newly formed oxygen anion **(25)** lead to the expulsion of the cyanide anion, a good nucleofuge, and ring closure (Scheme 6) [69]. 

However, it has been established that aurone formation may be avoided by providing milder conditions for the dehydrohalogenation reaction [64]. Donnelly and Doran have observed that the quantity of flavone increases with a decrease in base concentration [70]. von Auwers and Anschutz have shown that 4′-alkoxyflavones can be obtained by performing the cyclization reaction in cold alcohol rather than hot alcohol, which generates aurones [64,71].

Zemplén and Bognár improved the von Kostanecki method and demonstrated that nuclear bromination could be avoided by submitting acetates of hydroxyflavanones to bromination in absolute chloroform and in the presence of UV light. The obtained intermediate was a 3-bromoflavanone that could be easily dehydrobrominated, thus forming a flavone. This method is suitable for obtaining 3-hydroxyflavones [62,66].

Improvements in the direct dehydrogenation of chalcones and flavanones were made by using selenium dioxide as an oxidative reagent [66,72].

**Scheme 7:**
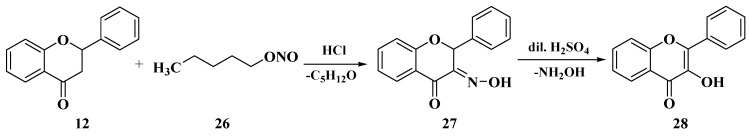
von Kostanecki–Szabránski method for obtaining 3-hydroxyflavones.

A new method by von Kostanecki was established in 1904 in collaboration with Szabránski. This method is used for obtaining 3-hydroxyflavones **(28)** from flavanones **(12)** via isonitrosoflavanones. The flavanone is nitrosated with pentyl nitrite **(26)** and hydrochloric acid. The isonitrosoflavanone **(27)** is converted into 3-hydroxyflavone **(28)** via hydrolysis with diluted sulfuric acid (Scheme 7) [73].

### 3.2. Von Auwers–Müller Method

**Scheme 8:**
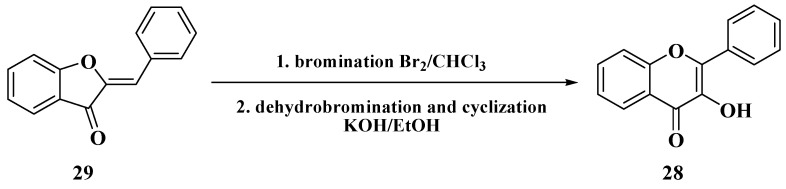
The von Auwers–Müller method of obtaining 3-hydroxyflavones.

**Scheme 9:**
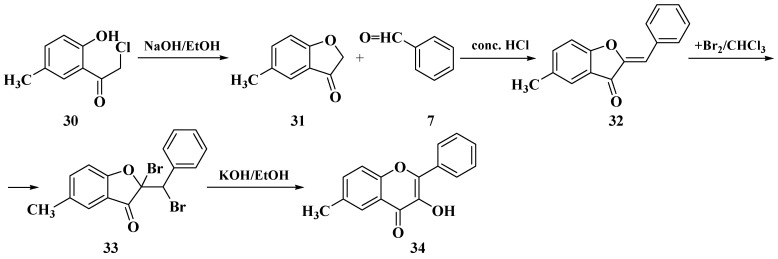
Original von Auwers synthesis using 5-methylcoumaranone [74].

Karl von Auwers’s method was first established in 1908 in collaboration with Müller, and it consists of the conversion of aurones **29** into 3-hydroxyflavones **28** (Scheme 8) via bromination in chloroform, followed by the dehydrohalogenation of the intermediate **(33)** with ring rearrangement under the action of potassium hydroxide in ethanol solution (Scheme 9) [3,74].

Originally, the aurone used by von Auwers and Müller was 2-benzylidene-5-methylcoumaranone **32**, obtained from 5-methylcoumaranone **31** (previously synthesized by Stoermer and Bartsch) [75]. Von Auwers and Müller synthesized coumaranone **31** starting from *o*-chloracetyl-*p*-cresol **(30)** (Scheme 9) [74]. This is supposed to lead to a cyclodehydrohalogenation via the action of sodium hydroxide in ethanol while heating. The obtained coumaranone **31**, via condensation with benzaldehyde, is converted into the aurone **32**, which can add bromine to the ethylene bond. The obtained dibromo derivative **33** is transformed into 3-hydroxyflavone **34** via dehydrobromination and recyclization under the action of potassium hydroxide in ethanol while heating [74].

**Scheme 10:**
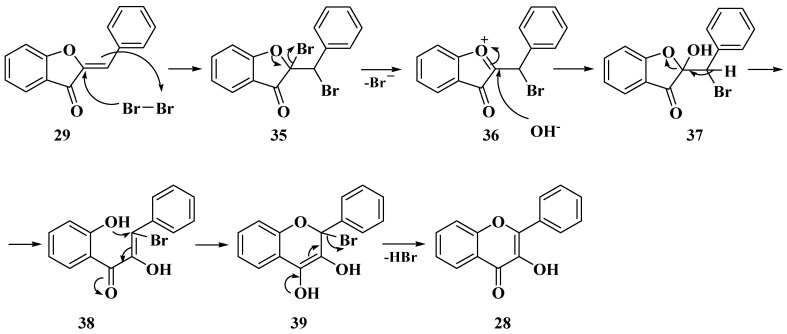
Mechanism of von Auwers synthesis [3].

The mechanism of von Auwers synthesis is presented in Scheme 10. It starts with the bromination of aurone on the double bond, resulting in a 1,2-dibrominated compound **(35)**. The subsequent substitution of bromine via the nucleophilic attack of a hydroxide anion results in an *α*,*β*-unsaturated ketone **(38)**, which yields hydroxyflavone through cyclodebromination [3].

As much as this method tried to improve upon von Kostanecki’s one, it also had limited applicability for obtaining natural 3-hydroxyflavones. To improve the outcome of this method, they suggested chlorination instead of bromination. This resulted in a trichlorinated derivative that would convert into a chlorinated hydroxyflavone that has one chlorine atom in positions 5 or 7. However, any attempt to eliminate the last chlorine atom failed. 3-Hydroxyflavones could be obtained from aurones in better yields only if the splitting of the coumaranone ring would take place easier than the dehydrohalogenation. They concluded that the presence of chlorine, methoxy, and methyl groups in position 5 of the coumaranone ring facilitates the formation of 3-hydroxyflavones, while methoxy and methyl groups in meta position and two methoxy groups on the aldehyde make it more difficult [76,77].

### 3.3. Allan–Robinson Method

**Scheme 11:**
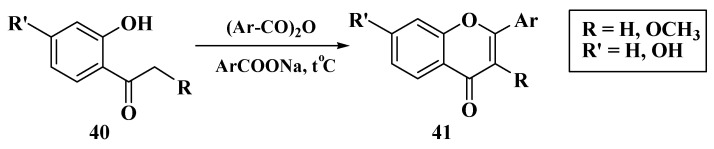
Allan–Robinson method for obtaining flavones.

The Allan–Robinson method was first established in 1924, and it involves converting 2-hydroxyacetophenones to flavones via treatment with anhydrides of aromatic carboxylic acids and the sodium salts of the corresponding carboxylic acids while heating (Scheme 11). The first part of this procedure entails converting *ω*-methoxyresacetophenone (Scheme 11: R′ = OH, R = OCH_3_) into 7-hydroxy-3-methoxyflavone **(41)** using benzoic anhydride and sodium benzoate (Ar = C_6_H_5_). This method was further extended by using various 2-hydroxyacetophenone derivatives **(40)** and aromatic anhydrides as starting materials [78].

**Scheme 12:**
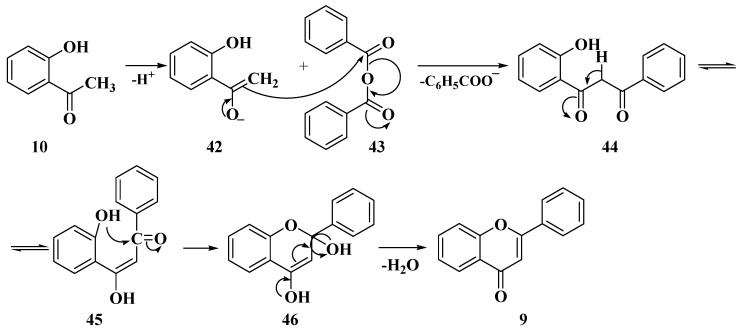
General reaction mechanism of the Allan–Robinson method for the synthesis of flavones.

In the first step of the reaction mechanism, the deprotonated enolic tautomer of 2-hydroxyacetophenone **(42)** performs a nucleophilic attack on the carbonyl group of the anhydride **(43)**, affording a 1,3-diketone compound (*o*-hidroxydibenzoylmethane) **(44)**. The basic conditions provided by the sodium salt transform the intermediate into a flavone via enolisation followed by intramolecular cyclocondensation (Scheme 12) [3].

**Scheme 13:**
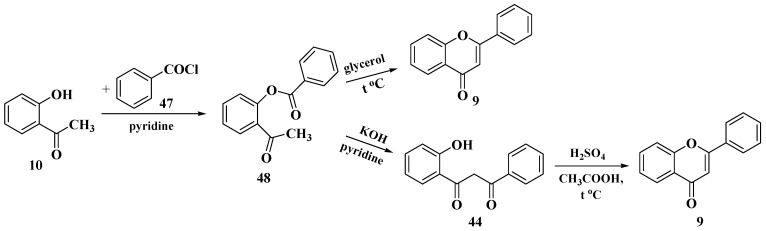
Wheeler’s alternative reaction conditions for the synthesis of flavones [79].

An alternative to the reaction conditions of this method was proposed by Wheeler. This modification implies that 2-hydroxyacetophenone **(10)** is turned into 2-benzoyloxyacetophenone **(48)** via treatment with benzoyl chloride and pyridine. The obtained acetophenone derivative **(48)** can be converted into a flavone **(9)** either in glycerol while heating or via treatment with KOH in pyridine, followed by the cyclization of the 1,3-diketone intermediate **(44)** with glacial acetic acid and concentrated sulfuric acid while heating (Scheme 13) [79].

Unlike the previous presented methods, this one can be used for synthesizing more complex structures, making the Allan–Robinson method suitable for obtaining natural flavones and 3-hydroxyflavones. It has been used for obtaining a large variety of natural compounds, including fisetin, quercitin [80], datiscetin [81], myricetin, methylgalangin [82], limocitrol, limocitrin, spinacitrin [83], kaempferol [84], axillarin [85], jaceidin [86], and hispidulin [8].

### 3.4. Baker–Verkataraman Method

**Scheme 14:**
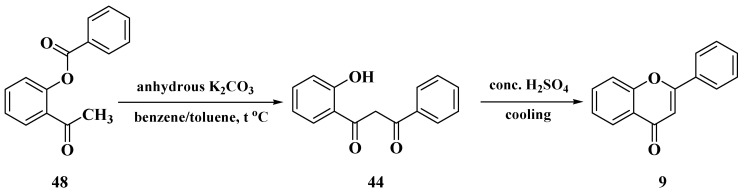
The Baker–Venkataraman method.

This method was established after the individual work of Baker and Verkataraman in 1933. It is used to obtain flavones from *o*-acyloxyacetophenones **(48)**. These precursors are first converted into *o*-hydroxydibenzoylmethane derivatives **(44)** via heating in benzene or toluene with anhydrous potassium carbonate. The *o*-hydroxydibenzoylmethane derivatives **(44)** are cyclized into the corresponding flavones **(9)** via treatment with cold concentrated sulfuric acid [3,87] (Scheme 14).

Verkataraman first used this method to obtain *α*-naphtoflavone from 2-acetyl-1-naphthyl benzoate via heating with sodium benzoate and benzoic anhydride [88,89]. Mahal and Verkataraman obtained the diketone derivative via treatment with NaNH_2_ in ether at room temperature. Further cyclization to the corresponding *α*-naphtoflavone was performed via treatment with concentrated sulfuric acid in ethanol at reflux [90].

**Scheme 15:**
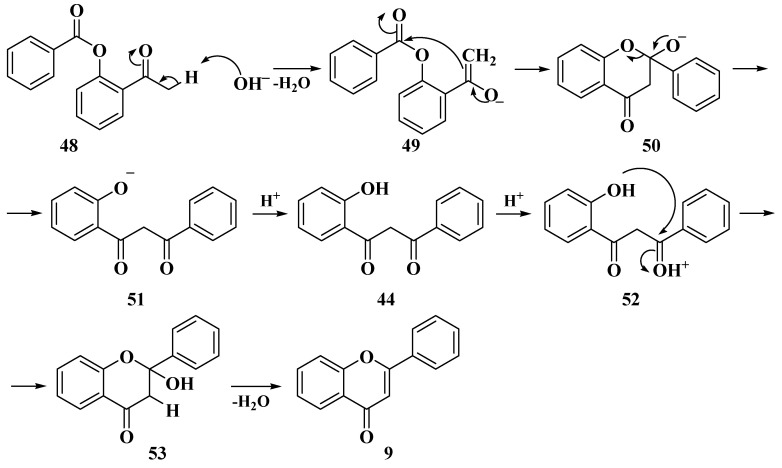
The mechanism of the Baker–Verkataraman method [3,91].

According to the mechanism, this method starts with an intramolecular Claisen condensation between acetophenone and an ester group grafted in ortho position on the aromatic ring (an *o*-acyloxyacetophenone **48**), which can also be interpreted as acyl group transfer. This is followed by cyclocondensation in acidic conditions via a 2-hydroxyflavanone intermediate **(53)** (Scheme 15) [3,91]. 

**Scheme 16:**
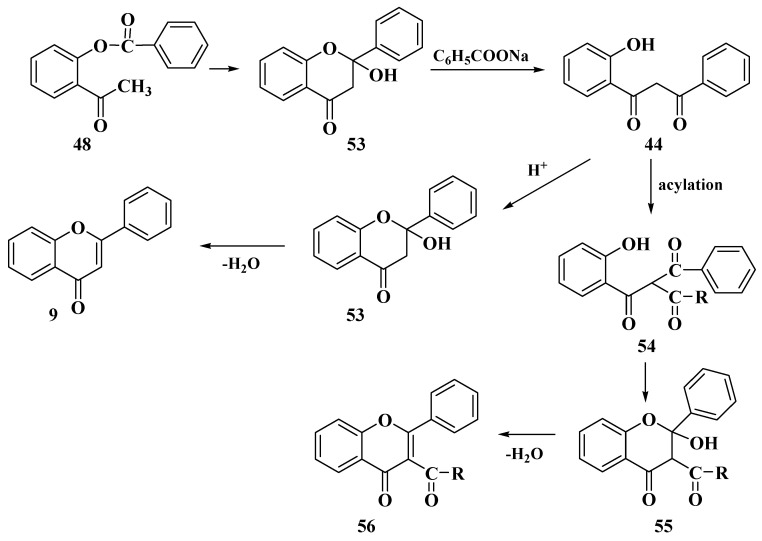
Synthesis of 3-acylflavones.

Baker found out that this method could yield 3-acylflavones **(56)** via the treatment of *o*-acyloxyacetophenone **48** with sodium salts of carboxylic acids. Instead of cyclocondensation, the *o*-hydroxydibenzoylmethane **44** can be acylated on the methylene carbon, affording a triacylmethane derivative **(54)**. This intermediate is cyclized to 2-hydroxy-3-acylflavanone **(55)** and then dehydrated, affording the corresponding 3-acylflavone **(56)** (Scheme 16) [87].

However, the conventional method could not produce large yields of flavones [91]. Cramer and Elschnig discovered that the best catalyst is sodium ethoxide [92]. Ares et al. suggested using potassium tert-butoxide for the synthesis of the diketone intermediate, obtaining higher yields [93]. Jain et al. used benzoyl chloride in benzene under phase transfer-catalysis conditions with *n*-tetrabutylamonium hydrogen sulphate, obtaining *o*-hydroxydibenzoylmethane. Further treatment with *p*-toluenesulphonic acid yielded flavones with good results [94]. Modifying this method permits the synthesis of hydroxyflavones with phloroglucinol units via heating the 2-hydroxyacetophenones with aqueous 5% potassium carbonate followed by treatment with acetic acid [95]. Song and Ahn proposed the use of tetrabutylammonium fluoride as a phase transfer catalyst for the condensation of dibenzoylmethanes, also obtaining good yields [96]. Another useful reaction condition for cyclocondensation was discovered by Stanek and Stodulski, who used *N*-triflylphosphoramide, an organocatalyst which is active in mild reaction conditions [97]. Through using microwave irradiation, Pinto et al. managed to obtain 3-aroyl-5-hydroxyflavones from 2,6-diaroyloxyacetophenones [98]. Similar results were obtained with a shorter reaction time by using ethyl ammonium nitrate, a recyclable ionic liquid, under microwave irradiation [99].

Through the cyclization of dibenzoylmethanes with CuBr_2_, 3-bromoflavones are formed, and these can then be converted into 3-aminoflavones [100]. Other catalysts that are reportedly useful for converting dibenzoylmethanes into flavones include the following: FeCl_3_ in dichloromethane [101]; CuCl_2_ under microwave irradiation [102]; Cobalt(bis(salicylideniminato-3-propyl)hydroxide, a six coordinate cobalt Schiff base complex [103]; montmorillonite K 10 Clay under microwave irradiation (clay-catalyzed synthesis) [104]; amberlyst 15, a cation-exchange resin, under reflux in isopropyl alcohol [105]; solid supported catalysts like mesoporous titania/tungstophosphoric acid composites TiO_2_/H_3_PW_12_O_40_ at reflux [106], trifluoromethanesulfonic acid in toluene at reflux [107], Wells–Dawson heteropolyacid in toluene at reflux (or solvent-free) [108], molybdophosphoric and molybdosilicic Keggin heteropolyacids in acetonitrile at reflux [109], and silica gel supported NaHSO_4_ in toluene at reflux [110].

### 3.5. Algar–Flynn–Oyamada Method

**Scheme 17:**
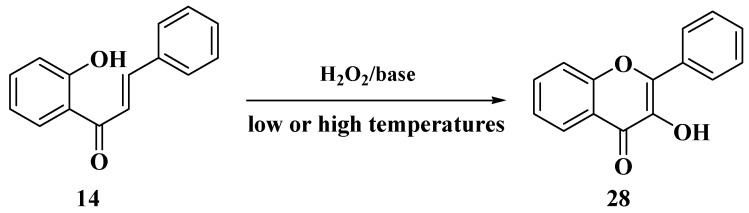
The Algar–Flynn–Oyamada method.

This method represents the collaboration between Algar and Flynn and the individual work of Oyamada from 1934 to 1935. It can be used to obtain 3-hydroxyflavones **(28)** from *o*-hydroxychalcones **(14)** by means of hydrogen peroxide in aqueous sodium hydroxide solution and cooling [111]. Algar and Flynn used this method with hot potassium hydroxide alcoholic solution, and both achieved good yields (Scheme 17) [112].

**Scheme 18:**
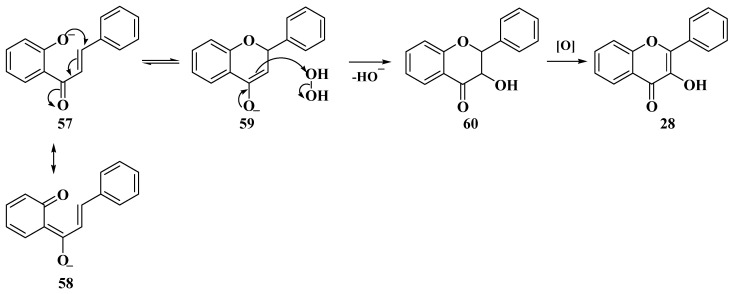
The mechanism of the Algar–Flynn–Oyamada method proposed by Dean and Podimuang.

The mechanism has experienced many alterations over time. At first, Algar and Flynn were not able to isolate the intermediates. They proposed the transitory formation of an ethylene peroxide in the first stage of oxidation [112]. Oyamada considered the existence of a flavanone intermediate **(60)** formed by the electrophilic attack of hydrogen peroxide on position 3 of the flavanone anion **(59)** [111,113]. Dean and Podimuang demonstrated that no epoxides were formed as intermediates for obtaining 3-hydroxyflavones **(28)**. They argued that a *β*-position attack by the hydroperoxide anion would be difficult for phenolic chalcones due to the internal electronic inactivation (see mesomere structure **58**) and the basic conditions that turn them into anions, facilitating the electrostatic repulsion of the hydrogen peroxide (Scheme 18) [114].

**Scheme 19:**
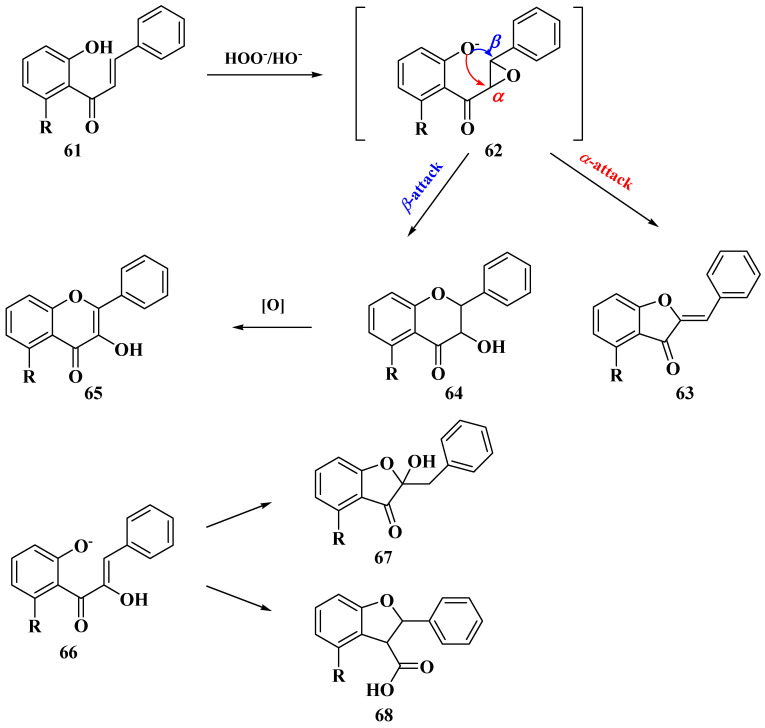
Algar–Flynn–Oyamada reaction products. Aurone formation happens in higher yields when a 6′-substituted chalcone is used.

Starting from *o*-hydroxychalcones, via epoxides as intermediates, two cyclization products can theoretically be formed, namely, aurones **(63)**, if the attack takes place in the *α* position, and flavonols **(65)** (via flavanone **64**), if the attack takes place in the *β* position (Scheme 19). When 6′-substituted-2′-hydroxychalcones **(61)** were used as precursors, it was found that the cyclization takes place preferentially via *α* attack, with the formation of aurones as major reaction products. This is due to the fact that the substituent grafted in the 6′ position of the chalcone displaces the keto group from the plane of the aromatic ring. This causes the steric inhibition of resonance from the 2’-oxygen anion and determines the activation of the *α*-carbon [77].

Adams and Main argued that we should not rule out the idea that epoxides are precursors and intermediates in the formation of flavonols via *β*-attack. They demonstrated that treating an epoxide at various pHs in aqueous acetonitrile solution and room temperature led to small amounts of the *β*-cyclization product, which was a flavonol derivative [115,116]. 

Dean and Podimuang’s theory was also challenged by Serdiuk et al. They concluded that epoxides are indeed intermediates in this method by analyzing the thermodynamic characteristics of the intermediate reactions and finding out that the reactions of chalcones in anionic form with the hydroperoxide anion are energetically favorable [113]. However, Bhattacharyya and Hatua demonstrated through the density functional theory that epoxidation is unlikely because of the electrostatic interaction of the hydroperoxide anion with the conjugated double bond, although an epoxide intermediate could still be formed at high temperatures and converted into aurone, supporting Dean and Podimuang’s work [117].

The major limitation of the Algar–Flynn–Oyamada method is the fact that it cannot be applied for the synthesis of 3-hydroxyflavones via the cyclization of the corresponding 6′-substituted *o*-hydroxychalcones because, in this case, cyclization takes place preferentially via *α*-attack [118]. Also, besides 3-hydroxyflavones, aurones can be easily obtained through *α*-cyclization, and sometimes, even 2-benzyl-2-hydroxydihydrobenzofuran-3-ones **(67)** and 2-arylbenzofuran-3-carboxylic acids **(68)** are formed [116,119] (Scheme 19).

Oxidative cyclization with H_2_O_2_/NaOH or KOH of *o*-hydroxychalcones was applied by Li Xiang et al. for the synthesis of new 3-*O*-substituted flavonols as anti-prostate cancer agents [32]. Khdera H.A. et al. recently synthesized a series of flavonol derivatives with antifungal properties via the oxidative cyclization of *o*-hydroxychalcones with H_2_O_2_/KOH, followed by the further derivatization of the 3-OH group [57].

A couple of modifications for this method exist, namely, using Na_2_CO_3_ and H_2_O_2_ in methanol and water to obtain 5′-substituted-3-hydroxyflavones [120], phase transfer catalysis (tetrabutylammonium bromide, iodide, or hydrogensulphate; benzyltriphenylphosphonium chloride; ethyltriphenylphosphonium iodide; propyltriphenylphosphonium iodide or bromide) [121], and direct synthesis starting from acetophenone and aldehyde without isolating the chalcone intermediate (known as one-pot synthesis) [55,122,123,124,125].

Other modern improvements have been made for the Algar–Flynn–Oyamada reaction, such as performing the synthesis under microwave irradiation [55].

**Scheme 20:**
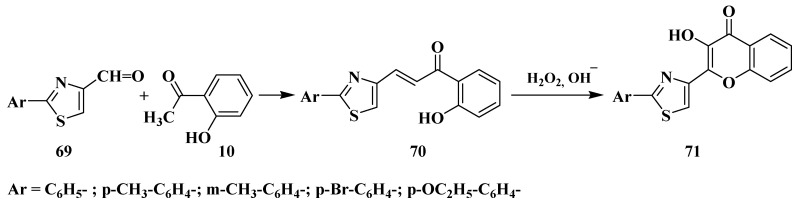
Synthesis of new thiazole hydroxychromones via the oxidative cyclization of thiazole *o*-hydroxy chalcones [126].

The oxidative cyclization of *o*-hydroxychalcones with H_2_O_2_/OH^−^ was successfully extended by our research group for the synthesis of new analogs of hydroxyflavones containing the 2-phenylthiazole moiety instead of the phenyl B ring of the basic skeleton of natural flavones. In the first step, the thiazole *o*-hydroxy-heterochalcones **(70)** were obtained in 75–82% yields via the condensation of *o*-hydroxyacetophenone **(10)** with different 2-arylthiazol-4-yl carbaldehydes **(69)** (Scheme 20). Their epoxidation with hydrogen peroxide, followed by oxidative cyclization, afforded the corresponding 2-arylthiazole hydroxyflavones **(71)** in 65–71% yields [126].

**Scheme 21:**
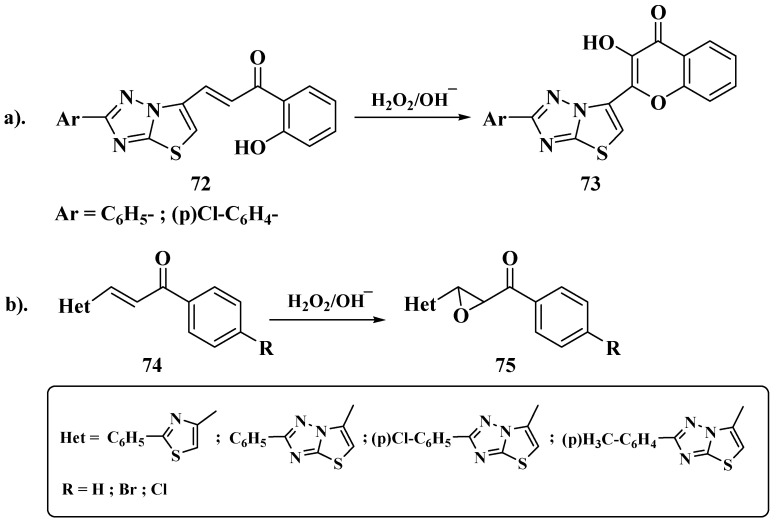
(**a**) Synthesis of thiazolo[3,2-*b*][1,2,4]triazole hydroxyflavones via the cyclization of *o*-hydroxychalcones. (**b**) Synthesis of thiazole and thiazolo[3,2-*b*][1,2,4]triazole epoxyketones [127].

The cyclization pathway of thiazole and thiazolo[3,2-*b*][1,2,4]triazole hetero-chalcones with hydrogen peroxide in basic media (NaOH) was investigated by V. Zaharia et al. Through treatment with the hydrogen peroxide of *o*-hydroxy-heterochalcones **(72)** in basic media, the corresponding hydroxyflavones **(73)** were obtained (Scheme 21a). The epoxyketones **(75)** were obtained in the same reaction conditions as the hydroxyflavones **(73)**, starting from the unhydroxylated heterochalcones **(74)** as precursors (Scheme 21b) [127].

### 3.6. Claisen–Schmidt Method

**Scheme 22:**
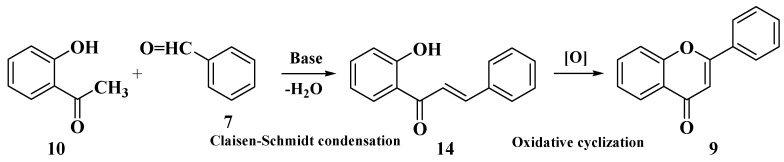
The Claisen–Schmidt method.

This method was established in 1962 and consists of two steps. The first step is Claisen–Schmidt condensation between an *o*-hydroxyacetophenone **(10)** and benzaldehyde derivative **(7)** in basic medium, which affords chalcones. The second step involves the oxidative cyclization of the obtained chalcones **(14)**, which can be achieved using a large variety of conditions and catalysts [3] (Scheme 22).

**Scheme 23:**
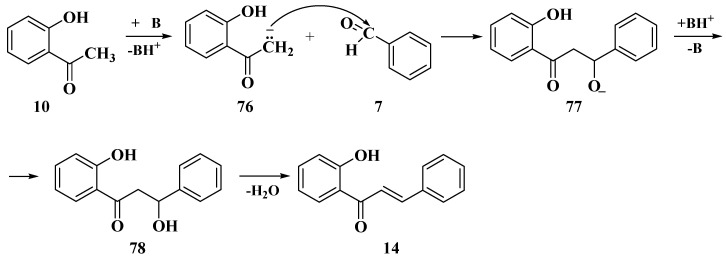
The mechanism of Claisen–Schmidt condensation [128].

The condensation mechanism begins with the formation of an anion of the acetophenone **(76)** under basic conditions. Through the nucleophilic attack of the anion **(76)** on the carbonyl group of benzaldehyde **(7)**, followed by the elimination of H_2_O, the corresponding chalcones **(14)** are obtained [128] (Scheme 23).

**Scheme 24:**
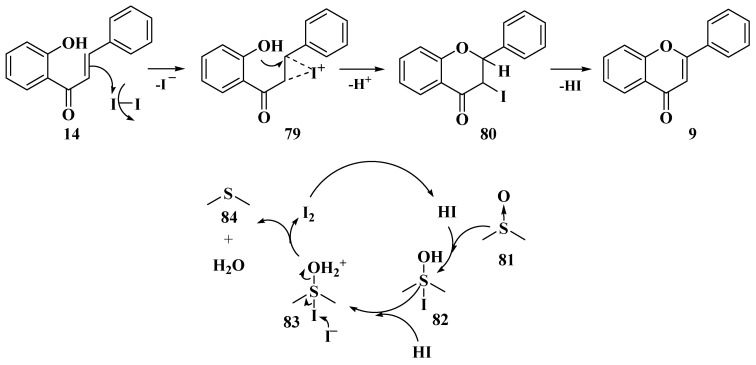
Mechanism of the oxidative cyclization of *o*-hydroxychalcones with I_2_/DMSO. Role of DMSO in the regeneration of iodine (mechanism adapted from Masesane) [130].

Cyclization can be realized by many methods, first starting with iodine in hot dimethyl sulfoxide (I_2_/DMSO, Δ). Patonay et al. observed that this method is suitable for a large variety of substituents, including electron-donating and electron-withdrawing groups and sensitive-to-oxidation and protecting groups. Thus, it can be considered a general method of cyclization for obtaining flavones from 2′-hydroxychalcones [129]. The mechanism involves the formation of an iodonium cation **(79)** via the interaction of I_2_ with the *o*-hydroxychalcone **(14)**, followed by cyclization via the nucleophilic attack of the *o*-hydroxy group. Further elimination of hydroiodic acid affords the corresponding flavones **(9)**. The solvent dimethyl sulfoxide **(81)** is important in this reaction because it acts as a co-oxidant and regenerates iodine [130] (Scheme 24). 

**Scheme 25:**
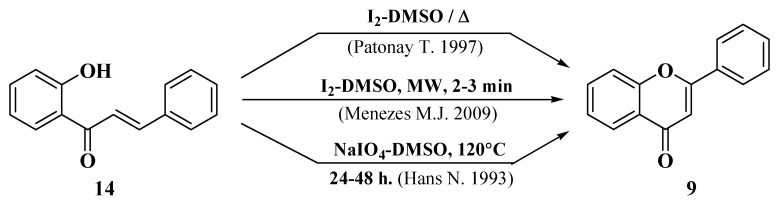
Oxidative cyclization of *o*-hydroxychalcones using I_2_ or NaIO_4_ in DMSO [129,131,132].

Through using microwave irradiation, the reaction time is greatly reduced to approximatively three minutes [131]. However, this method is limited in the case of 2′-hydroxychalcones with a phloroglucinol moiety, affording complex mixtures. Hans and Grover extended the applicability of iodine as an oxidant agent by instead using sodium periodate in hot dimethyl sulfoxide (NaIO_4_/DMSO, Δ), managing to smoothly convert phloroglucinol-derived chalcones into the corresponding flavones [132] (Scheme 25).

**Scheme 26:**
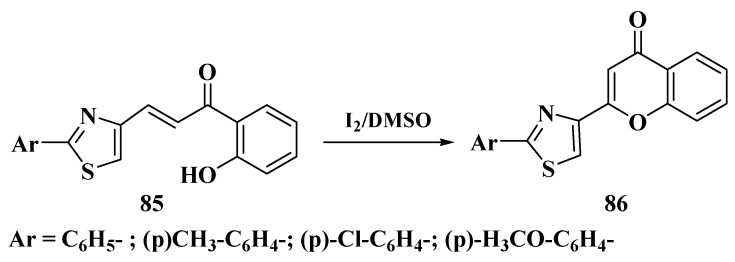
Cyclization of 2-arylthiazole *o*-hydroxychalcones with iodine in dimethyl sulfoxide [133].

In order to extend the general method of cyclization of *o*-hydroxychalcones with iodine in dimethyl sulfoxide, our research group investigated this method using a series of 2-arylthiazole *o*-hydroxychalcones as precursors **(85)**. A similar chemical behavior was observed in this case, with the corresponding 2-arylthiazole flavones **(86)** being obtained with 32–55% yields (Scheme 26) [133].

At present, the cyclization of *o*-hydroxychalcones using iodine in dimethyl sulfoxide still represents the principal method for the synthesis of flavones and their analogs with various structures. This method is still finding multiple applications because it generally allows for obtaining the target compounds with high yields, while the *o*-hydroxychalcones needed as precursors can be easily obtained via Claisen–Schmidt condensation [30,34,35,46,47,61].

**Scheme 27:**
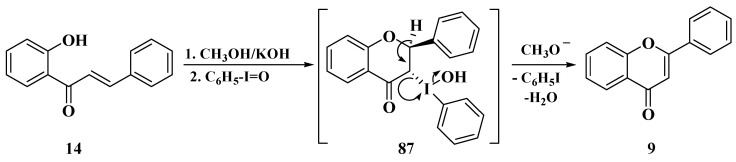
Oxidative cyclization using hypervalent iodine reagent [134].

Another method for the cyclization of *o*-hydroxychalcones was established by Litkei et al., who used iodosobenzene diacetate (phenyliodine(III) diacetate, PIDA), a hypervalent iodine reagent, which forms iodosylbenzene in situ [134] (Scheme 27). The same reagent was used for obtaining prenylated flavones, which are abundant in nature, from prenylated 2′-hydroxychalcones [135].

**Scheme 28:**
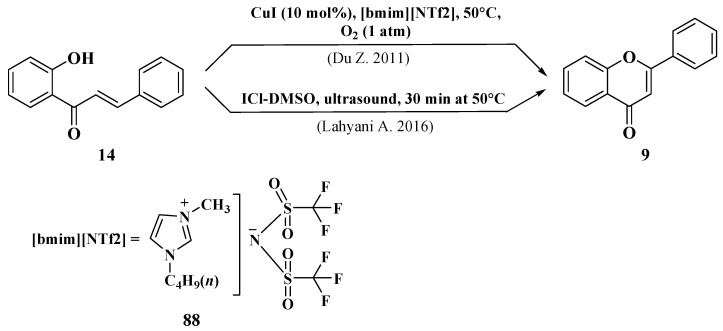
Oxidative cyclization of *o*-hydroxychalcones with CuI in ionic liquid [136] and ICl in DMSO, respectively, under ultrasound [137].

The usage of ionic liquids for this oxidative cyclization was described in the literature. Du et al. developed a new method involving Cu(I) iodide, mediated by the ionic liquid [bmim][NTf2] (1-butyl-3-methylimidazolium bis(trifluoromethanesulfonyl)imide, compound **88**) at a low temperature. The reaction mechanism has not been fully elucidated, but the results so far reveal that a flavanone is formed intermediately. The flavanone seems to be dehydrogenated to the corresponding flavone in the same reaction conditions [136]. Lahyani and Trabelsi reported the oxidative cyclization of *o*-hydroxychalcones with iodine monochloride in dimethyl sulfoxide (ICl-DMSO) under ultrasound. This method has similar advantages to than of ultrasound processes, such as mild conditions, high yields, and eco-friendliness. The mechanism is similar to the one based on oxidative cyclization with I_2_-DMSO [137] (Scheme 28).

**Scheme 29:**
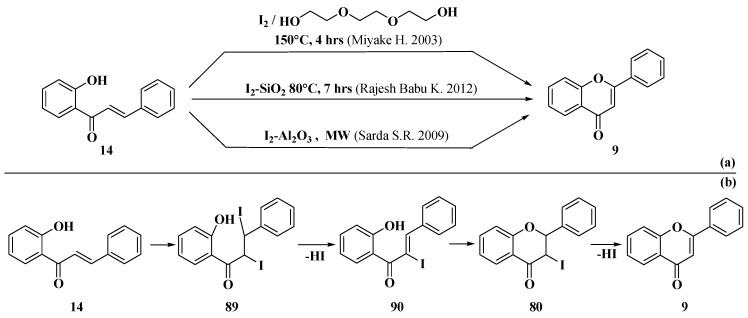
(**a**) Oxidative cyclization with iodine in triethylene glycol and solid supports (silica gel and alumina) [138,139,140]. (**b**) The proposed pathway for oxidation with molecular iodine (adapted from Miyake et al. [138]).

Heating *o*-hydroxychalcones with iodine in triethylene glycol is also a good and inexpensive method for their cyclization. While the underlying mechanism is not fully understood, the authors proposed a pathway that involves the iodination of chalcone, which affords chalcone diiodides **(89)**, similar to the chalcone dihalides from the von Kostanecki’s method. Via the dehydrohalogenation of the intermediate **(90)**, a 3-iodoflavanone **(80)** is formed, which yields the corresponding flavone **(9)** via *β*-elimination of a second hydroiodic acid molecule [138]. The use of iodine on silica gel (I_2_-SiO_2_) was reported by Babu et al. to provide favorable results and less harmful effects towards the environment [139]. Another solid supported catalyst, iodine on neutral alumina (I_2_-Al_2_O_3_), was reported by Sarda et al., providing short reaction times, simple conditions, and very good yields [140] (Scheme 29).

**Scheme 30:**
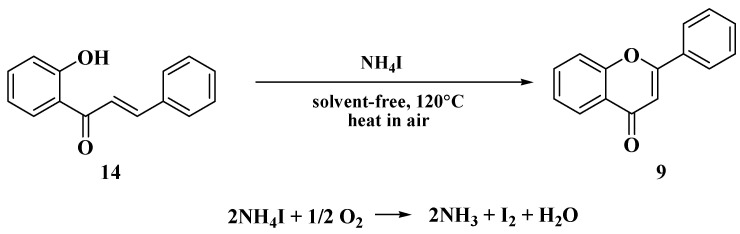
Oxidative cyclization using NH_4_I in solvent-free conditions [141].

An alternative to the toxicity and corrosiveness of iodine was proposed by Kulkarni et al., who used ammonium iodide under exposure to air and solvent-free conditions, thus generating in situ iodine that acted as a cyclization agent [141] (Scheme 30).

**Scheme 31:**
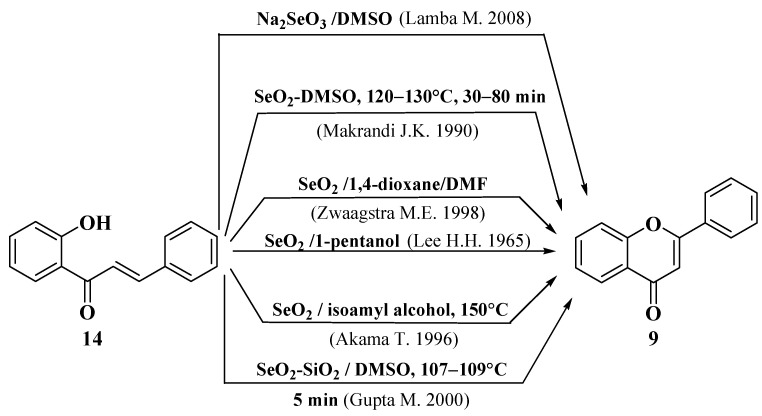
Oxidative cyclization of *o*-hydroxychalcones using selenium-based catalysts in different solvents and reaction conditions [142,143,144,145,146,147].

Selenium dioxide is another catalyst used for the cyclization of *o*-hydroxychalcones. It can be combined with various solvents, such as pentan-1-ol [142] (only providing low yields) [132], dioxane [143], and isoamyl alcohol with prolonged heating, to facilitate the formation of side products and low yields [144]. Dimethyl sulfoxide was also used with good yields [145], but in order to diminish the high toxicity of DMSO, Gupta et al. managed to use selenium dioxide and traces of solvent over silica gel under microwave irradiation, yielding very good results [146]. Similar to I_2_/DMSO, SeO_2_/DMSO is also problematic for chalcones with a phloroglucinol oxygenation pattern [132]. However, selenium dioxide is volatile and hazardous. Lamba and Makrandi proposed using sodium selenite (Na_2_SeO_3_), which is less volatile, and found out that it acts as a proper dehydrogenating agent in DMSO [147] (Scheme 31).

**Scheme 32:**
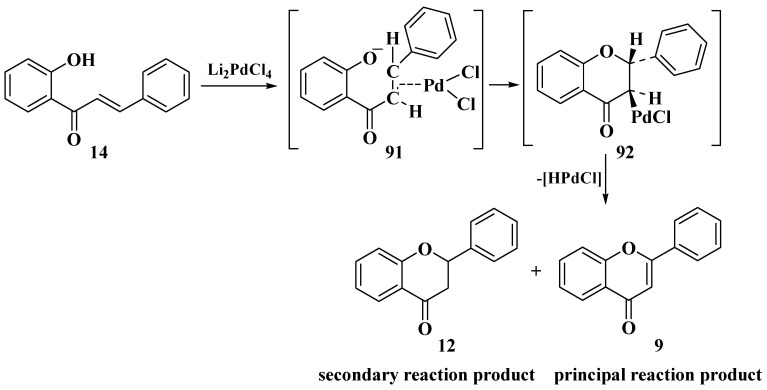
Oxidative cyclization of *o*-hydroxychalcones with palladium(II) salts [148].

Palladium was also experimented on as a catalyst. Kasahara et al. used lithium chloropalladite (Li_2_PdCl_4_), palladium(II) acetate, and (CH_3_COO)_2_Pd to obtain flavones. The reaction was described as a phenoxypalladation, with the formation of the intermediates **91** and **92**, followed by the elimination of palladium (II) hydride (HPdCl). This method also yielded small amounts of flavanone **(12)** [148] (Scheme 32).

**Scheme 33:**
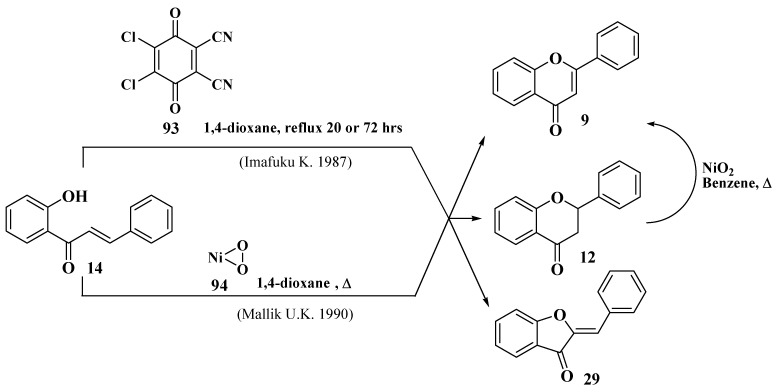
Oxidative cyclization of *o*-hydroxychalcones using DDQ and nickel peroxide [149,150].

Cyclodehydrogenation with DDQ (2,3-dichloro-5,6-dicyano-p-benzoquinone) was proposed by Imafuku et al. Their method involves using dioxane as a solvent and results in a mixture of flavones, flavanones, and aurones in low yields [149]. Another agent that acts in a similar manner is nickel peroxide (NiO_2_) in dioxane, yielding a similar mixture. However, flavanones can be dehydrogenated to flavones under the action of NiO_2_ in benzene as a solvent [150] (Scheme 33).

**Scheme 34:**
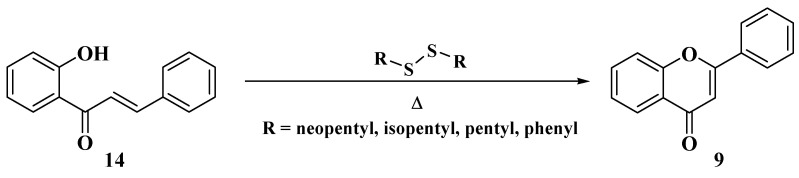
Oxidative cyclization using organic disulfides.

Disulfides are also a good choice for cyclodehydrogenation. Hoshino et al. used four disulfides, namely, dineopentyl disulfide, diisopentyl disulfide, dipentyl disulfide, and diphenyl disulfide, with the latter giving the best yields. The disadvantages of this method include the very high temperatures (260–290 °C) and very low yields when electron-withdrawing groups (NO_2_) are present [151] (Scheme 34).

**Scheme 35:**
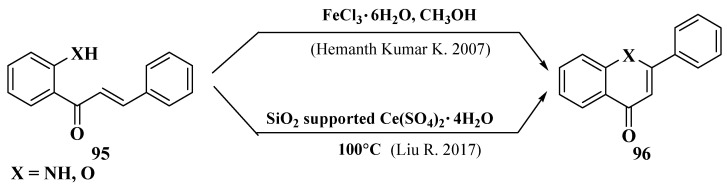
Oxidative cyclization of *o*-hydroxychalcones using FeCl_3_ hexahydrate or Ce(SO_4_)_2_ tetrahydrate [152,153].

Initially meant for obtaining quinolines from 2′-aminochalcones using FeCl_3_·6H_2_O in methanol, Kumar and Perumal applied the same method on 2′-hydroxychalcones and obtained flavones (**96**, X = O) with satisfactory results [152]. Similarly, Liu et al. used cerium(IV) sulphate tetrahydrate (Ce(SO_4_)_2_·4H_2_O) on silica gel to obtain flavones from 2′-hydroxychalcones at 100 °C, aza-flavones (**96**, X = NH), and aza-flavanones from 2′-aminochalcones (**95**, X = NH) [153] (Scheme 35). 

**Scheme 36:**
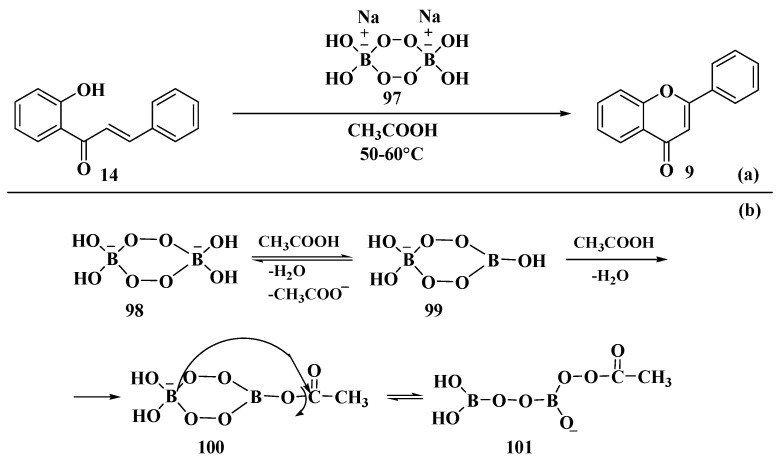
(**a**) Oxidative cyclization of *o*-hydroxychalcones using sodium perborate tetrahydrate (SPB). (**b**) The mechanism for generating peracetoxyboron anionic species [154,155].

In addition to all the reagents previously mentioned, sodium perborate tetrahydrate (SPB, compound **97**) was proposed by Ganguly et al. They observed that depending on the solvent, this method could yield different products, such as warm acetic acid and SPB in excess yielded flavones **(9)**, while warm aqueous acetonitrile yielded flavanones. In the case of flavone formation, SPB and acetic acid generate peracetoxyboron anion species **(101)** that favorize the oxidative cyclization of *o*-hydroxychalcones [154,155] (Scheme 36).

**Scheme 37:**
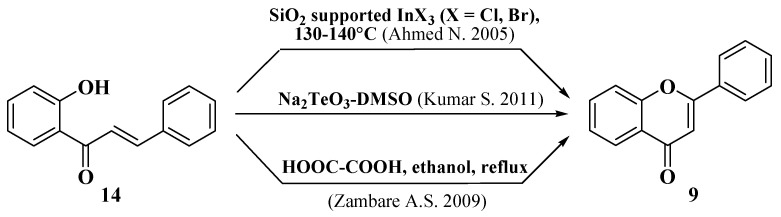
Oxidative cyclization of *o*-hydroxychalcones with indium(III) halides [156], sodium tellurite in DMSO [157], and oxalic acid [158].

Other reagents for the oxidative cyclization include indium(III) halides (InCl_3_ and InBr_3_) on silica gel and in solvent-free conditions, which has been shown to provide higher yields when InBr_3_ is used [156] or when sodium tellurite is used in dimethyl sulfoxide and anhydrous conditions (Na_2_TeO_3_-DMSO) [157]. The reaction mechanism has not completely ben elucidated. The supposed reported pathway for the oxidative cyclization with indium halides involves the formation of a flavanone as an intermediate, which is dehydrogenated in the same reaction conditions to the corresponding flavone [156]. Oxalic acid in ethanol reflux, which was used by Zambare et al., has been shown to be a very useful and cheap method, with excellent yields over 90% [158] (Scheme 37).

**Scheme 38:**
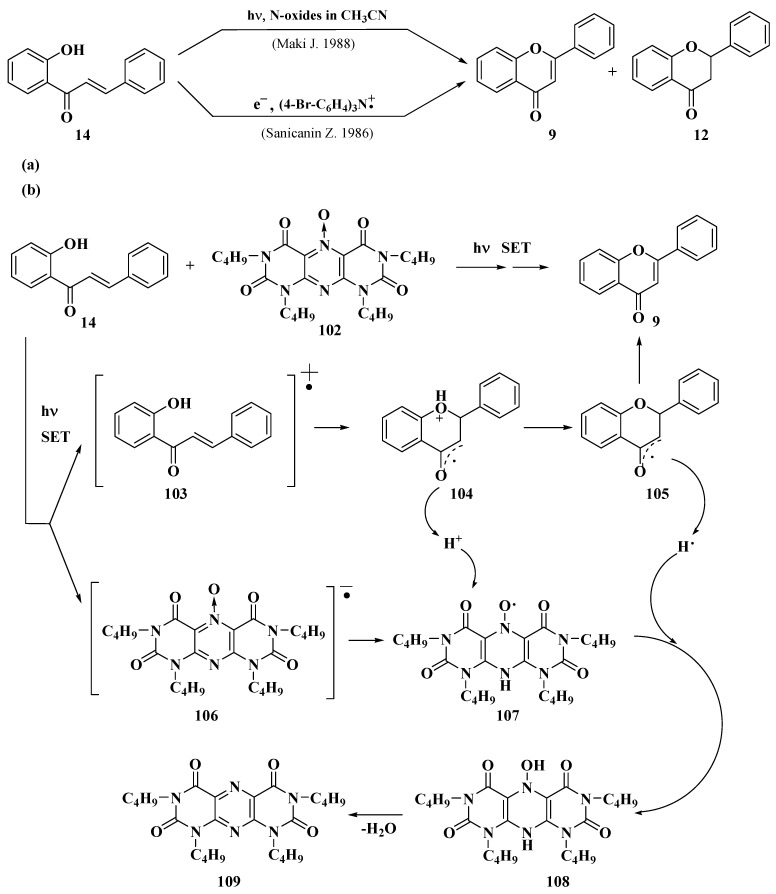
(**a**) Oxidative cyclization using photo- and electrochemistry [160,161]. (**b**) Mechanism of single-electron transfer process (adapted from Maki et al. [160]).

Photocyclization provides another way to perform this reaction. However, in one study, it yielded only flavanones and in low quantities [159]. By adding a heterocyclic *N*-oxide, pyrimido[5,4-g]pteridine *N*-oxide **(102)**, Maki et al. managed to obtain flavones **(9)** but still only in unsatisfactory yields and in a mixture with flavanones **(12)**. The photoreaction involves a single electron transfer (SET) process from chalcone **(14)** to *N*-oxide **(102)**. Initially, the *N*-oxide is found in the oxygenated form, pyrimido [5,4-g]pteridin-2,4,6,8(1*H*,3*H*,7*H*,9*H*)-tetrone 5-oxide **(102)**, and as the mixture forms, it gets deoxygenated to pyrimido [5,4-g]pteridine **(109)** [160]. Electrochemistry found applications in this reaction too. Saničanin and Tabaković cyclized 2′-hydroxychalcones by electrochemically generating a cation radical of tris-(4-bromophenyl)amine, which acted as a homogenous electron transfer reagent. This method creates a mixture of flavanones and flavones in moderate yields [161] (Scheme 38).

**Scheme 39:**
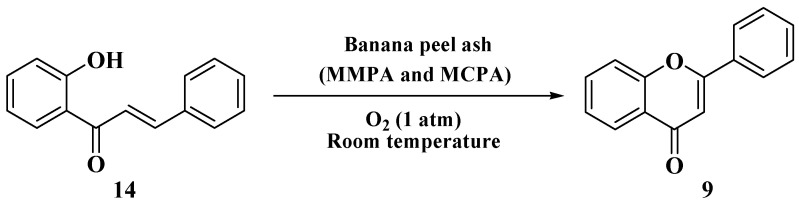
Oxidative cyclization using banana peel ash in open air and solvent-free conditions (adapted from Tamuli et al. [162]).

Through creating a totally non-hazardous medium, a different approach for the cyclization of *o*-hydroxychalcones was implemented by Tamuli et al. In this case, the catalyst consists of a mixture of two agro-food waste products—*Musa* sp. ‘Malbhog’ peel ash (MMPA) and *Musa champa* Hort. ex Hook. F. peel ash (MCPA)—which allowed for the cyclodehydrogenation of *o*-hydroxychalcones in solvent-free conditions and at room temperature [162] (Scheme 39).

Among the reported methods for the cyclization of *o*-hydroxychalcones, our research group investigated the most promising ones in order to obtain new flavonoid analogs containing the 2-arylthiazole moiety instead of the benzene ring B. Our aim was to also investigate the chemical behavior of 2-arylthiazole *o*-hydroxychalcones in the cyclization reactions.

**Scheme 40:**
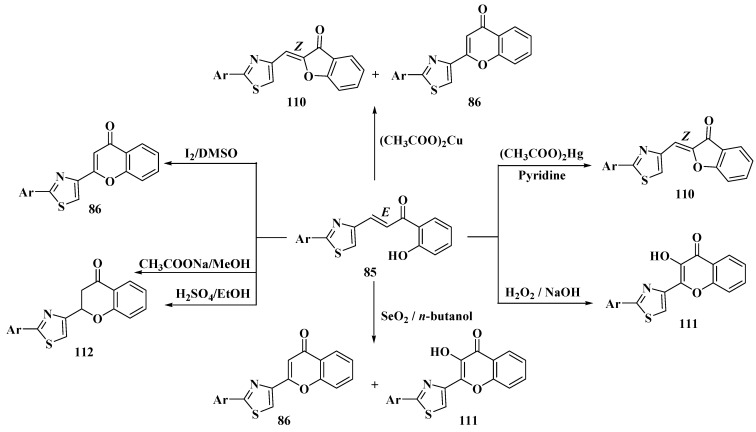
Cyclization products obtained via the oxidative cyclization of 2-arylthiazole *o*-hydroxychalcones in different reaction conditions [43,133,163].

The oxidative cyclization of the 2-arylthiazole *o*-hydroxychalcones **(85)** afforded various reaction products, depending on the oxidizing agent. Flavanones **(112)** were obtained with 40–55% yields via the cyclization of the corresponding 2-arylthiazole *o*-hydroxychalcones in acidic catalysis (H_2_SO_4_ conc. in ethanol) [133]. The cyclization of 2-arylthiazole *o*-hydroxychalcones in the presence of sodium acetate in methanol (used as a solvent) also afforded the corresponding flavanones **(112)** in good yields. The use of copper(II) acetate in dimethyl sulfoxyde resulted in a mixture of aurones **(110)** and the corresponding flavones **(86)** in an approximate 1:1 molar ratio [43]. Hydroxyflavones **(111)** were obtained via the cyclization of the same substrates with hydrogen peroxide in alkaline catalysis [43], and flavones **(86)** were obtained when iodine in dimethyl sulfoxide was used [43,133]. The cyclization with selenium dioxide in *n*-butanol led to a mixture of flavones **(86)** and hydroxyflavones **(111)** [43]. Cyclization with mercury(II) acetate in pyridine as a solvent afforded the corresponding *Z*-aurones **(110)** with 70–86% yields [43]. The cyclization products and the reactions conditions are summarized in Scheme 40.

**Scheme 41:**
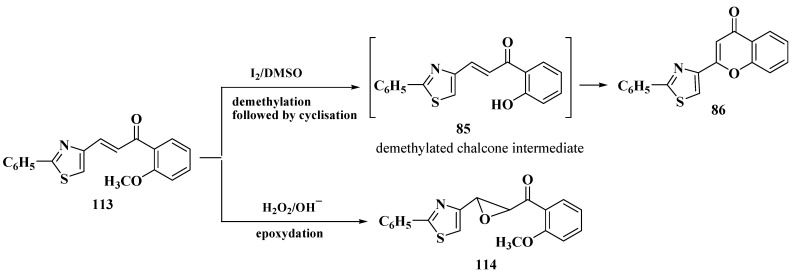
Cyclization products of 2-arylthiazole *o*-methoxychalcones [163].

The cyclization of *o*-methoxylated chalcones bearing the 2-arylthiazole moiety **(113)** was further studied in similar reaction conditions. It was found that the reaction occurred differently depending on the oxidizing agent and the reaction conditions. Through treatment with iodine in dimethyl sulfoxide, at reflux, the corresponding flavones **(86)** were formed. This fact indicates that the demethylation of the methoxy group of chalcone occurred, resulting in the corresponding *o*-hydroxychalcone **(85)**, which was further cyclized to the corresponding flavone [163]. Instead, when hydrogen peroxide in NaOH was used as a cyclization agent, the formation of the corresponding epoxides **(114)** was observed, which can be explained by the fact that the methoxy group is resistant in these reaction conditions; therefore, the cyclization to flavone cannot take place (Scheme 41) [163].

### 3.7. Mentzer Method

**Scheme 42:**
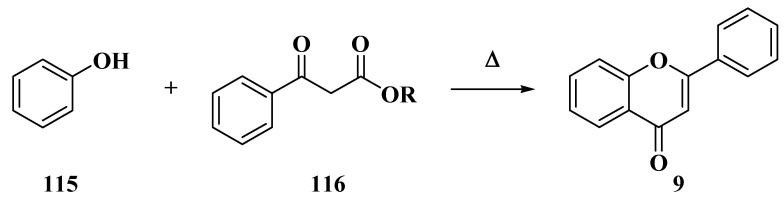
The Mentzer method for the synthesis of flavones.

This method involves synthesizing flavones based on the reaction between phenols (phenol, resorcinol, or phloroglucinol) and *β*-ketoesters **(116)** [164] (Scheme 42). It is based on the Pechmann reaction (used to obtain coumarins from phenol and ethyl acetoacetate) [165]. Mentzer et al. obtained flavones from resorcinol and ethyl 2-benzylacetoacetate by heating up the mixture for 48 h at 250 °C [166].

**Scheme 43:**
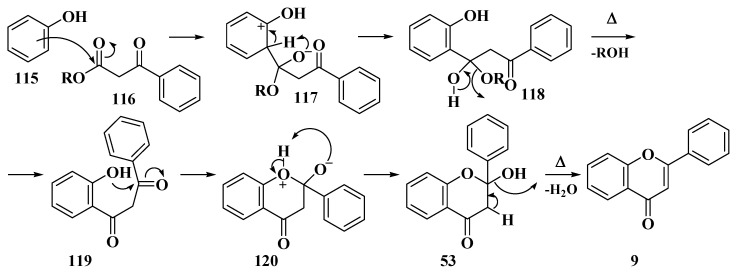
The mechanism of the Mentzer method (image adapted from Wang [164]).

The mechanism involves a nucleophilic attack by the phenolic compound **(115)** on the *β*-ketoester **(116)**, resulting in an arenium ion (**117)**. Under heating, the intermediate eliminates the alcohol and affords *o*-hydroxydibenzoylmethane **(119)**, a compound which is also available in the Baker–Verkataraman method, which is cyclized into flavone **(9)** [164] (Scheme 43).

**Scheme 44:**
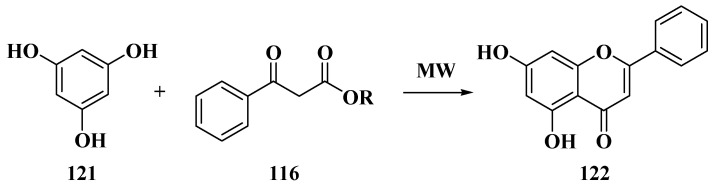
A variation of the Mentzer method using microwave irradiation (image adapted from Seijas et al. [167]).

Seijas et al. developed a solvent-free modification of this method that uses microwave irradiation instead of heating. The yields are good, and the time of reaction is significantly reduced [167] (Scheme 44).

### 3.8. Suzuki–Miyaura Method

**Scheme 45:**
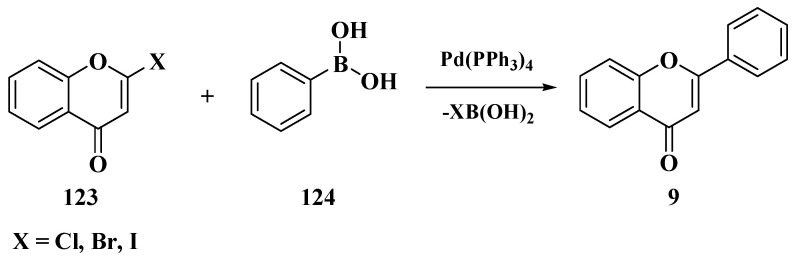
The Suzuki–Miyaura method for the synthesis of flavones starting from 2-halogenochromones [168,169].

The Suzuki–Miyaura method is a cross-coupling reaction based on the insertion of palladium in sp^2^ hybridized C-X bonds and the usage of various organoboron precursors **(124)** under mild reaction conditions. In the case of flavones, 2-halogenochromones **(123)** are used as substrates; however, they are difficult to obtain [168] (Scheme 45).

While this method mostly uses 2-bromo- or 2-iodochromones, Kraus and Gupta demonstrated that these precursors yield aurones rather than flavones. By using 2-chlorochromone, they managed to obtain flavones in proper yields (68–74%) [169].

**Scheme 46:**
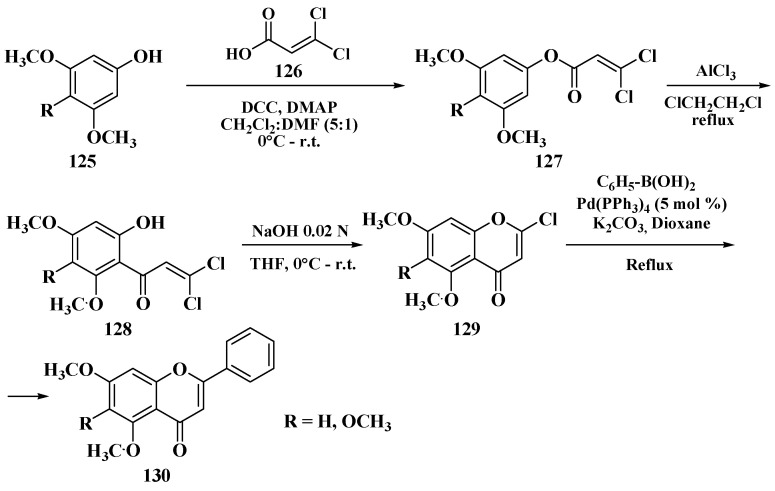
The pathway of the Suzuki–Miyaura method for obtaining substituted flavones (image adapted from Kraus and Gupta [169]).

Their synthesis starts with the esterification of a phenolic compound **(125)** and 3,3-dichloroacrylic acid **(126)**, resulting in a dichloro acrylic ester **(127)**. This compound undergoes Fries transposition, affording the intermediate **128**. Further cycloelimination in basic conditions yields 2-chlorochromone **(129)**. Finally, this is then coupled with arylboronic acids, which can replace the halogen atom from the chromone skeleton with the aryl rest of the boronic compound, thus affording a flavone **(130)** [169] (Scheme 46).

**Scheme 47:**
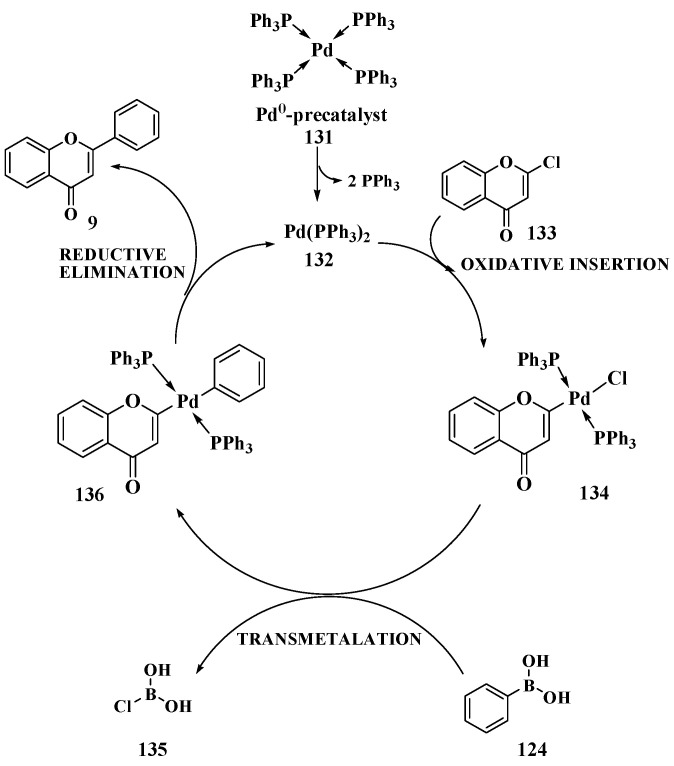
Postulated mechanism of the Suzuki–Miyaura method.

The postulated mechanism of the Suzuki–Miyaura method involves a catalytic cycle initiated by the formation of the active Pd catalytic species Pd(PPh_3_)_2_ **(132)**. Further oxidative insertion of palladium to the 2-halogenochromone **(133)** leads to the organopalladium intermediate **134** (chromon-2-yl-palladium(II) chloride). In the transmetalation step, the chlorine atom is transferred to the boronic compound **124**, resulting in a chloroboronic acid **(135)** and chromon-2-yl-phenyl-palladium **(136)**, which undergoes reductive elimination, yielding the flavone **(9)** and the active Pd catalyst Pd(PPh_3_)_2_ [170] (Scheme 47).

## 4. Chemical Synthesis of Aurones

**Scheme 48:**
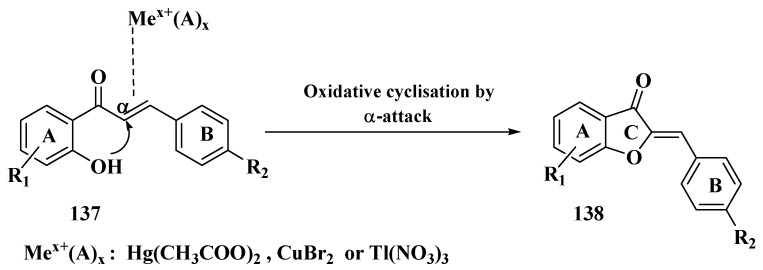
Aurone synthesis via the cyclization of *o*-hydroxychalcones [171,172,173].

Among the reported methods for the synthesis of aurones, the most applied are those based on the oxidative cyclization of *o*-hydroxychalcones. These reactions are mediated by transitional metal salts such as Hg(CH_3_COO)_2_ [40,48,171], CuBr_2_ [171,172], or Tl(NO_3_)_3_ [173], whose metal cation interacts with the double bond in chalcones, thus favoring the attack of the ortho hydroxy group on the alpha carbon (Scheme 48) [130].

The cyclization of *o*-hydroxychalcones **(137)** with mercury(II) acetate afforded the best yields in aurones **(138)** when the reaction was performed in pyridine [40,48,171] or with dimethyl sulfoxide [174] as a solvent at reflux. In the case of cyclization with cupric bromide, aprotic polar solvents such as dimethyl sulfoxide [171] or *N*,*N*-dimethyl formamide [37,172] have been shown to be the optimal solvents.

In the case of thallium nitrate-mediated cyclization, reported data indicate that the reaction course depends on the nature of the substituents on the B ring; only the electron-withdrawing groups (chlorine, formyl, methoxycarbonyl, and nitro) grafted in the para position are favorable for cyclization to aurones. In other cases, mixtures of aurones and isoflavones, or exclusively isoflavones, are obtained [175].

**Scheme 49:**
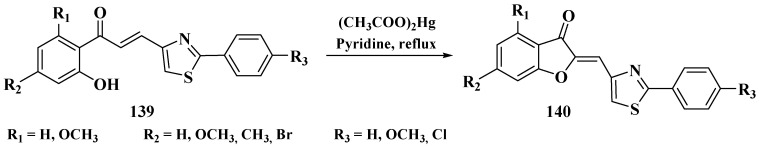
Synthesis of aurone analogs containing the 2-phenylthiazole moiety [43].

Our research group synthesized a series of aurone analogs **(140)** containing the same 2-phenylthiazole aromatic system as the B ring in *Z*-configuration, achieving 70-86% yields via the cyclization of the corresponding *o*-hydroxychalcones **(139)** with mercury(II) acetate in pyridine at reflux (Scheme 49) [43].

**Scheme 50:**
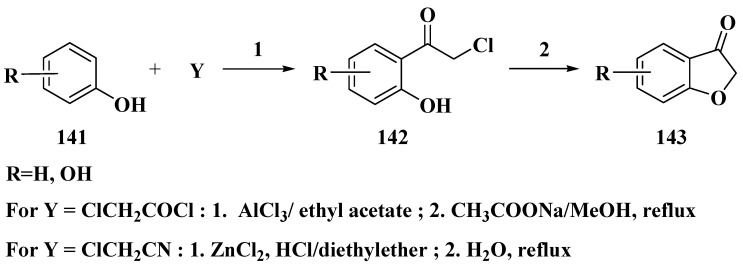
Synthesis of benzofuran-3-ones.

Another chemical route towards aurones involves starting with substituted benzofuran-3-ones as precursors. Benzofuran-3-ones **(143)** are synthesized in two steps. The first step consists of the introduction of a chloroacetyl group on the aromatic ring of the phenols **(141)** by applying a Hoesch-type reaction (with 2-chloronitriles in the presence of anhydrous ZnCl_2_ and gaseous HCl) [176] or Friedel–Crafts acylation (with alfa-halogenated acyl chlorides in the presence of AlCl_3_) [49,177]. In the next step, an intramolecular Williamson-type reaction with ring closure is applied (Scheme 50) [49,176,177].

**Scheme 51:**
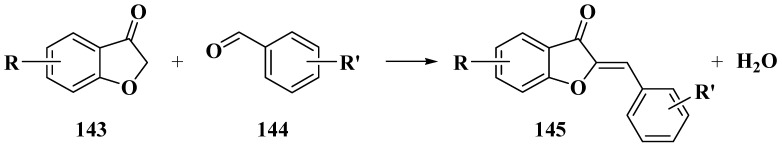
Synthesis of aurones via the condensation of aromatic aldehydes with benzofuran-3-ones.

The condensation of benzofuran-3-ones **(143)** with aromatic aldehydes **(144)** affords the corresponding aurones **145** (Scheme 51). This step can be performed under acid catalysis with HCl [52] and HCl/CH_3_COOH [49,74], in basic catalysis with 50% KOH/Ethanol or KOH/Methanol [176,177,178] or NaOH/NaOCH_3_ [50], or with a Al_2_O_3_ catalyst [75]. A recently reported method for the condensation of benzofuran-3-ones with aromatic aldehydes uses an activated Ba(OH)_2_ catalyst in dimethyl sulfoxide as a solvent [53,58].

**Scheme 52:**
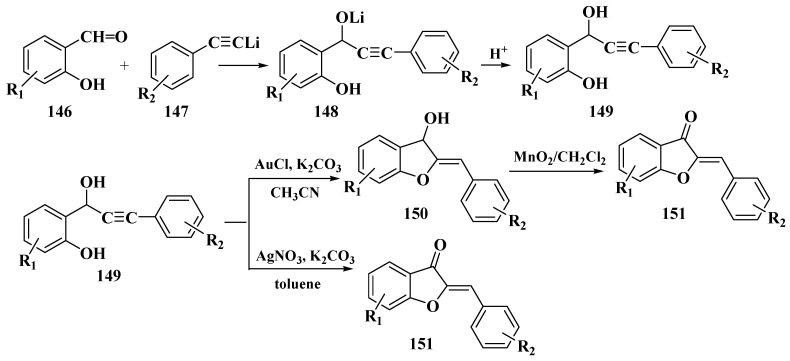
Synthesis of aurones via hydroxypropynylphenol derivatives [179,180].

Another reported method for the synthesis of aurones involves the cyclization of hydroxypropynylphenol derivatives **(149)** obtained via the alkynylation of salicylaldehyde derivatives **(146)** with lithium arylacetylures **(147)** (Scheme 52) [179,180].

Harkat H. et al. performed the cyclization of hydroxypropynylphenol derivatives **(149)** in the presence of gold(I) chloride and potassium carbonate in acetonitrile. Further oxidation with MnO_2_ of the intermediate secondary alcohol **(150)** afforded the corresponding aurones **151** (Scheme 52) [179].

Li S. et al. proposed a variant of cyclization with AgNO_3_. Ag^+^ ions catalyze both the cyclocondensation reaction and the oxidation of the secondary alcohols to the corresponding aurones **151** (Scheme 52) [180].

## 5. Conclusions

From a pharmacological point of view, flavones, flavonols, and aurones are precious natural products that have inspired researchers over time to create new biologically active compounds for use as anticancer and anti-infectious agents with benzochromon-4-one or benzofuran-3-one structures. 

The polyphenolic structure of natural flavones and aurones determines their low stability to oxidants in solution and also generally decreases their bioavailability after oral administration due to their low solubility, thus affecting their potential use as therapeutic agents. Consequently, the structural modulations on aromatic rings A and B that have been reported to aid the design of new flavone/aurone analogs and facilitate both the improvement of therapeutic properties and pharmacokinetic profiles and increases in stability must be taken into account.

The hybridization of the basic skeletons of flavones and aurones with other pharmacophore moieties has led to new compounds with superior pharmacological profiles compared to their natural analogs. 

The most applied routes for the synthesis of flavones, hydroxyflavones, and aurones involve the cyclization of *o*-hydroxychalcones. As exemplified in Section 3, the cyclization pathway transpires differently depending on the reaction conditions, the catalysts used, and the nature and position of the substituents grafted on the aromatic moieties [118,127].

In order to obtain new biologically active compounds based on the flavone scaffold, two main directions can be outlined, offering future research perspectives. One research direction involves using natural flavones already recognized for their therapeutic potential as precursors. Through the alkylation of their OH groups [10,45] or by grafting different pharmacophore units on their aromatic rings [31], new flavonoid analogs or hybrid molecules such as Mannich bases [56] with improved biological functions can be obtained. Based on literature data regarding the discovery of new important pharmacophore moieties targeting tumor-associated structures or recognized for their antimicrobial properties, new multi-target-acting hybrid molecules with anticancer or antimicrobial potential could be developed using similar strategies that involve starting from the structures of natural flavones.

Another research direction with important future prospects in the development of new flavone analogs involves the synthesis of the 2-phenyl-chromen-4-one system via different procedures. The most applied and promising route consists of the cyclization of *o*-hydroxychalcones with I_2_/DMSO [30,46,47,61,133]. Although this method was implemented long time ago, this procedure is still largely exploited and extended on various structures because it generally allows one to obtain target compounds with high yields and because the *o*-hydroxychalcones needed for use as precursors are accessible and easily obtainable via Claisen–Schmidt condensation.

The oxidative cyclization of *o*-hydroxychalcones with H_2_O_2_ in basic media represents a promising route with future prospects for the synthesis of 3-hydroxyflavones. The cyclization pathway transpires differently depending on the nature and the position of the substituents grafted on the aromatic moieties, thus affording 3-hydroxyflavones or aurones [116,118,119]. Modern improvements have been made to this method, such as performing the synthesis under microwave irradiation [55].

Even if the presented methods for the synthesis of flavones and aurones generally refer to obtaining the basic skeleton for natural flavones and aurones, in which the aromatic moieties are benzene and benzochromon-4-one or benzofuran-3-one rings, these methods have also been successfully applied in obtaining other new synthetic analogs of flavones or aurones containing other aromatic moieties such as heteroaromatic systems [35,43,53,55,58,61]. In particular, flavone/aurone analogs bearing the heterocyclic moieties thiazole, pyrazole, thiophene, pyridine, and quinoline instead of ring B represent basic skeletons with future prospects for the design and development of new heterocyclic flavonoid analogs with anticancer and antimicrobial potential.

The results presented in this review regarding the biological activity of flavones and related compounds confirm that the general synthetic pathways towards flavones, flavonols, and aurones have great importance for future research on the development of new effective anticancer and antimicrobial agents.

## Data Availability

Not applicable.
